# Relationships of RNA Polymerase II Genetic Interactors to Transcription Start Site Usage Defects and Growth in *Saccharomyces cerevisiae*

**DOI:** 10.1534/g3.114.015180

**Published:** 2014-11-06

**Authors:** Huiyan Jin, Craig D. Kaplan

**Affiliations:** Department of Biochemistry and Biophysics, Texas A&M University, College Station, Texas 77843

**Keywords:** transcription initiation, GTFs, gene expression, transcription start site, RNA polymerase, trigger loop

## Abstract

Transcription initiation by RNA Polymerase II (Pol II) is an essential step in gene expression and regulation in all organisms. Initiation requires a great number of factors, and defects in this process can be apparent in the form of altered transcription start site (TSS) selection in *Saccharomyces cerevisiae* (Baker’s yeast). It has been shown previously that TSS selection in *S. cerevisiae* is altered in Pol II catalytic mutants defective in a conserved active site feature known as the trigger loop. Pol II trigger loop mutants show growth phenotypes *in vivo* that correlate with biochemical defects *in vitro* and exhibit wide-ranging genetic interactions. We assessed how Pol II mutant growth phenotypes and TSS selection *in vivo* are modified by Pol II genetic interactors to estimate the relationship between altered TSS selection *in vivo* and organismal fitness of Pol II mutants. We examined whether the magnitude of TSS selection defects could be correlated with Pol II mutant-transcription factor double mutant phenotypes. We observed broad genetic interactions among Pol II trigger loop mutants and General Transcription Factor (GTF) alleles, with reduced-activity Pol II mutants especially sensitive to defects in TFIIB. However, Pol II mutant growth defects could be uncoupled from TSS selection defects in some Pol II allele-GTF allele double mutants, whereas a number of other Pol II genetic interactors did not influence *ADH1* start site selection alone or in combination with Pol II mutants. Initiation defects are likely only partially responsible for Pol II allele growth phenotypes, with some Pol II genetic interactors able to exacerbate Pol II mutant growth defects while leaving initiation at a model TSS selection promoter unaffected.

Pol II is essential for expression of all protein-coding genes, and determining how the combined defects of Pol II activity mutants in all steps of the Pol II cycle (*i.e.*, initiation, elongation, termination, cotranscriptional events) lead to growth defects is a difficult task. Initiation, the first step in transcription, is highly conserved, with regulation requiring a great number of factors (Hahn 2004; Cramer *et al.* 2008). Classical biochemical experiments using model promoters have shown that Pol II requires general transcription factor (GTF) TFIID, TFIIB, TFIIF, TFIIE, TFIIH for promoter recognition, formation of the preinitiation complex (PIC), promoter melting, and transcription start site (TSS) selection. The integration of these and other factors determines the efficiency of any particular promoter and the sequences that will be used to initiate transcription. Much remains to be understood about the functions of GTFs and how they integrate with Pol II activity during initiation. The effects of Pol II activity and roles of GTFs in initiation are especially visible in the process of TSS selection in *S. cerevisiae*, where most promoters use multiple start sites, and this usage is sensitive to a number of factors. Therefore, examination of mutant effects on TSS selection allows a window to the initiation process *in vivo*.

The most well-known core promoter element, the TATA box (consensus TATAWAWR motif in *S. cerevisiae*), is highly conserved throughout evolution but is only found at a subset of promoters. TSS selection at TATA element-dependent promoters in *S. cerevisiae* involves recognition of transcription start sites positioned 40–120 nucleotides (nt) downstream from the TATA box and the use of multiple start sites at most promoters, whether they are classified as TATA-containing (contains consensus TATA box) or not (Li *et al.* 1994; Dvir 2002; Basehoar *et al.* 2004; Corden 2008) (H. Jin and C. D. Kaplan, unpublished observations). Such extensive downstream positioning of TSSs in yeast is distinct from other eukaryotes for TATA-dependent promoters, where starts are more tightly focused ∼30 nt downstream of the beginning of the TATA box. Despite this difference in TSS distance to promoter element, in *S. cerevisiae* promoter melting appears to start 20–30 nt downstream of the TATA box and thus is similar to higher eukaryotes even though start sites can be more than 100 nt further downstream in *S. cerevisiae* (Giardina and Lis 1993). These results suggested that *S. cerevisiae* Pol II scans for favorable TSSs subsequent to promoter melting and open complex formation (reviewed in Kaplan 2013). A directional model for TSS scanning is strongly suggested from mutational analysis of start site regions and the distribution of TSSs when efficient start sites are compromised (Kuehner and Brow 2006; Kostrewa *et al.* 2009). The contribution of scanning to TSS usage in other organisms is unknown; however, the majority of promoters in higher eukaryotes utilize multiple, dispersed TSSs in a manner at least superficially analogous to *S. cerevisiae* (Choi *et al.* 2002; Hoskins *et al.* 2011; FANTOM Consortium *et al.* 2014; Haberle *et al.* 2014).

GTFs TFIIB (encoded by *SUA7* in *S. cerevisiae*) and TFIIF (encoded by *TFG1*, *TFG2*, and *TFG3* in *S. cerevisiae*) contribute to TSS selection. TFIIB bridges the TATA binding protein (TBP)-promoter DNA complex and Pol II, and likely stabilizes open complex formation through interacting with single-stranded DNA sequences in the PIC; TFIIF guides and stabilizes Pol II binding during assembly of PIC and appears to functionally interact with TFIIB, and may directly regulate Pol II activity (Henry *et al.* 1994; Sun and Hampsey 1995; Hampsey 1998; Chen and Hampsey 2004; Chen and Hahn 2004; Eichner *et al.* 2010; Luse 2012). A number of *tfg1* and *tfg2* alleles have been shown to shift distribution of TSSs toward upstream positions (Ghazy *et al.* 2004; Freire-Picos *et al.* 2005; Majovski *et al.* 2005; Khaperskyy *et al.* 2008; Eichner *et al.* 2010; Hahn and Young 2011). Conversely, mutations in *SUA7* generally have been shown to alter TSS distribution toward downstream positions (Pinto *et al.* 1992, 1994; Hull *et al.* 1995; Sun and Hampsey 1995; Wu *et al.* 1999; Faitar *et al.* 2001; Chen and Hampsey 2004). Combination of TFIIB and TFIIF alleles can confer mutual suppression of their respective TSS defects along with TFIIF alleles’ suppression of TFIIB alleles’ temperature-sensitive phenotypes (Sun and Hampsey 1995; Ghazy *et al.* 2004; Freire-Picos *et al.* 2005). In addition, alleles of *SSL2*, which encodes an ATPase/helicase enzymatic subunit of TFIIH, have been shown to shift distribution of TSSs toward upstream slightly, and one allele that was shown to partially suppress downstream TSS shifts the cold sensitivity of *sua7-1*, suggesting functional importance of Ssl2 in TSS selection (Goel *et al.* 2012). Alleles of some Pol II subunits have been shown to affect TSS usage distribution on their own and that of GTF alleles when combined. Combination of alleles in *tfg1* and *rpo21/rpb1* resulted in suppressed temperature sensitivity and TSS defects of an *rpo21/rpb1* allele (Freire-Picos *et al.* 2005). Combination of a *tfg1* allele and *rpb9∆* resulted in exacerbated TSS defects and temperature sensitivity (Ghazy *et al.* 2004). Alleles of *rpb2* and *rpb9* were shown to suppress the downstream shift effect and cold temperature sensitivity of certain *sua7* alleles (Sun and Hampsey 1996; Sun *et al.* 1996; Chen and Hampsey 2004). Thus, for some GTF alleles, effects on TSS selection parallel their effects on growth phenotypes when combined with *rpb* alleles.

Our characterization of the relationship between Pol II catalytic activity mutants and TSS defects suggested an activity-based framework for interpretation of Pol II mutant TSS defects (Kaplan *et al.* 2012; Braberg *et al.* 2013). A mechanistic explanation for the connection of Pol II activity and TSS selection will be critical for understanding Pol II initiation. Mutations in Pol II subunit-encoding genes *RPO21/RPB1*, *RPB2*, *RPB7*, and *RPB9* have been previously shown to alter TSS utilization *in vivo*, however, the mechanism of the alteration has been unclear (Hull *et al.* 1995; Sun and Hampsey 1996; Sun *et al.* 1996; Chen and Hampsey 2004; Freire-Picos *et al.* 2005; Chen *et al.* 2007; Kaplan *et al.* 2012; Braberg *et al.* 2013). In previous work, it was shown that *rpo21/rpb1* mutants with substitutions in the trigger loop (TL), a mobile portion of the Pol II active center, have altered elongation rates *in vitro*. One class of these Pol II mutants confers faster elongation rates [termed gain of function (GOF)] *in vitro*, another class confers slower elongation rates [termed loss of function (LOF)], and the two classes are generally mutually suppressive when combined. These Pol II catalytic activity mutants conferred various phenotypes both *in vitro* and *in vivo*, including altered TSS selection, RNA splicing, presumed termination or processing defects, and chromosome segregation defects (Kaplan *et al.* 2008; Kireeva *et al.* 2008; Kaplan *et al.* 2012; Larson *et al.* 2012; Braberg *et al.* 2013; Viktorovskaya *et al.* 2013). The severity of TSS defects *in vivo* in both GOFs and LOFs correlated well with the extent of their deviation from WT elongation rate *in vitro*.

We previously found that Pol II GOF mutants shifted the distribution of TSSs upstream at *ADH1* and other genes, similarly to *tfg2* alleles and *rpb9∆*; conversely, Pol II LOF mutants shifted distribution of TSSs downstream at *ADH1*, similarly to most *sua7* alleles (Kaplan *et al.* 2012; Braberg *et al.* 2013). Directional alteration of *ADH1* TSS distribution by Pol II mutants, both GOFs and LOFs, mimic their effects on TSS distributions genome wide (H. Jin and C. D. Kaplan, unpublished results). Just as with the severity of their TSS defects, these Pol II mutants have growth defects *in vivo* that correlate with the extent of Pol II activity alteration. Mutants that have more severely altered activity *in vitro* (both fast and slow) show greater growth defects, more genetic interactions, and greater alterations to gene expression profiles *in vivo*. Growth defects can be suppressed when Pol II LOF and GOF mutations are combined within the same enzyme; similarly, there is mutual suppression of Pol II mutant TSS distribution defects at *ADH1* in the double mutant, indicating a correlation between TSS defects and general growth defects (Kaplan *et al.* 2012). Because most Pol II mutant phenotypes we have studied correlate with strength of observed biochemical defects, it is difficult to distinguish whether observed *in vivo* growth defects derive especially from defects in a particular facet of transcription. Through genetic experiments and examination of TSS selection *in vivo*, we have attempted to understand further the relationship between Pol II activity defects, GTF function, and transcription initiation in *S. cerevisiae*. We also set out to extend our previous studies of factors that genetically interact with Pol II (genetic interactors) (Braberg *et al.* 2013) to understand their roles in TSS selection and the relationship between initiation and growth defects of Pol II alleles.

## Materials and Methods

### Yeast strains and media

Plasmids containing *tfg2∆146-180*, *tfg2∆261-273*, *tfg2∆233-248* alleles were gifts from Steve Hahn (Eichner *et al.* 2010), plasmids containing *sua7-1*, *sua7-3* were gifts from Michael Hampsey (Chen and Hampsey 2004). The *sua7-58A5* and *sua7-70A5* alleles were generated by Quickchange site-directed mutagenesis according to directions of the manufacturer (Stratagene/Agilent). Fragments containing target alleles were cloned into the yeast integrating vector pRS306 (Sikorski and Hieter 1989) and transformed into a strain background used for phenotyping and primer extension assay. For the complete list of yeast and bacterial strains used in this study, see Supporting Information, Table S1. Please see Supporting Information for note on the *rpb1* mutant N1082S used in these studies.

Yeast media used in phenotyping assays were made as previously described (Amberg *et al.* 2005; Kaplan *et al.* 2012; Braberg *et al.* 2013). YP media contained yeast extract (1% w/v; BD), peptone (2% w/v; BD), and bacto agar (2% w/v; BD) supplemented with adenine and tryptophan. YPD media contained dextrose (2% w/v, VWR), YPRaf media contained raffinose (2% w/v, Amresco), and YPRafGal media contained raffinose (2% w/v) and galactose (1% w/v; Amresco) as the carbon source. YPRaf and YPRafGal media also contained antimycin A (1 µg/ml; Sigma-Aldrich). Synthetic complete media were made with “Hopkins mix” with certain amino acids dropped out at the concentrations described in Kaplan *et al.* (2012) after the slight modifications of Amberg *et al.* (2005). SC-Leu+MPA media contains 20 µg/ml final concentration of mycophenolic acid (Sigma-Aldrich) from a 10 mg/ml concentrated stock in ethanol (stored at −20°).

Mycophenolic acid (MPA) lowers cellular concentration of GTP by inhibition of IMPDH activity and induces expression of *IMD2*, which encodes an MPA-resistant form of *IMPDH*. Transcription mutants that are sensitive to lower GTP levels or those defective in induction of *IMD2* confer MPA sensitivity (MPA^s^). For Pol II trigger loop mutants, MPA sensitivity is predictive of upstream start site defects at *ADH1* (Braberg *et al.* 2013).

Strains used in this study contain the *lys2-128∂* allele (Simchen *et al.* 1984) that renders cells auxotrophic for lysine due to a Ty1 retroelement long terminal repeat (LTR) insertion in the 5′ end of *LYS2*. Mutants that alter transcription at the allele can grow on medium lacking lysine (*e.g.*, SC-Lys), a phenotype referred to as Spt^−^ (Suppressor of Ty). The *gal10∆56* allele (Greger *et al.* 2000; Kaplan *et al.* 2005) comprises a deletion in the *GAL10* 3′-UTR, resulting in compromised RNA processing and termination at *GAL10*, allowing transcription readthrough downstream into *GAL7*. Lack of *GAL7* gene product allows accumulation of toxic intermediate products in galactose metabolism, thus WT *gal10∆56* cells are sensitive to presence of galactose in the medium when *GAL* genes are expressed even in the presence of an additional usable carbon source (*e.g.*, YPRafGal). Mutants that alter readthrough from *gal10∆56* or otherwise increase *GAL7* expression show galactose resistance on YPRafGal media, a phenotype referred to as Gal^R^.

### Primer extension assay for start site utilization detection

Primer extension assays were performed as previously described (Ranish and Hahn 1991) and with a few modifications described in (Kaplan *et al.* 2012). Briefly, 30 µg total RNA purified as previously described (Schmitt *et al.* 1990) was used to anneal with ^32^P-labeld oligonucleotide priming downstream of *ADH1* start sites in 15 µl total reaction volume. Reverse-transcription reaction was performed by M-MLV reverse-transcriptase (Fermentas) in the presence of RNase Inhibitor (Fermentas) in 45 µl total reaction volume. Products were precipitated, digested with RNase A, and separated in 8% acrylamide gel made with 19:1 acrylamide:bisacrylamide (Bio-Rad), 1XTBE, and 7M urea, followed by visualization by phosphorimaging (Bio-Rad) and quantification with ImageQuant 5.1 software (GE).

### Heatmaps for genetic interaction phenotypes

Growth on each media were scored using a 0–5 scoring system (0 = no growth, 5 = WT growth for all media except YPRafGal and SC-Lys; 0 = WT growth, 5 = growth of the mutant with maximum growth on the corresponding plate for YPRafGal and SC-Lys). To indicate growth difference in different growth conditions, all mutants on YPD, YPD 37°, YPRaf, SC-Leu phenotypes were normalized to WT on each plate by subtraction (mutant score−WT score). Negative numbers (slower growth) are shown as blue, positive numbers (faster growth) are shown in red, and inviable double mutants are in dark gray. Growth differences on SC-Leu+MPA were normalized to growth difference on SC-Leu by subtracting the difference on SC-Leu from the difference on SC-Leu+MPA, thus rendering net growth difference due to MPA sensitivity/resistance. Differences on YPRafGal and SC-Lys (WT growth is zero) were normalized to growth difference on YPD and SC-Leu (“standard growth condition” controls for these phenotyping media) by dividing the difference on YPRafGal or SC-Lys by ratio of WT growth to mutant growth on YPD or SC-Leu to quantify resistance phenotypes. Mutants that have Gal^R^ or Spt^−^ phenotypes are thus shown as red in the heatmaps. Calculated score difference tables were turned into heatmaps using GENE-E (http://www.broadinstitute.org/cancer/software/GENE-E/index.html).

## Results

### Allele-specific genetic interactions between GTF mutants and Pol II trigger mutants

**Figure 1:**
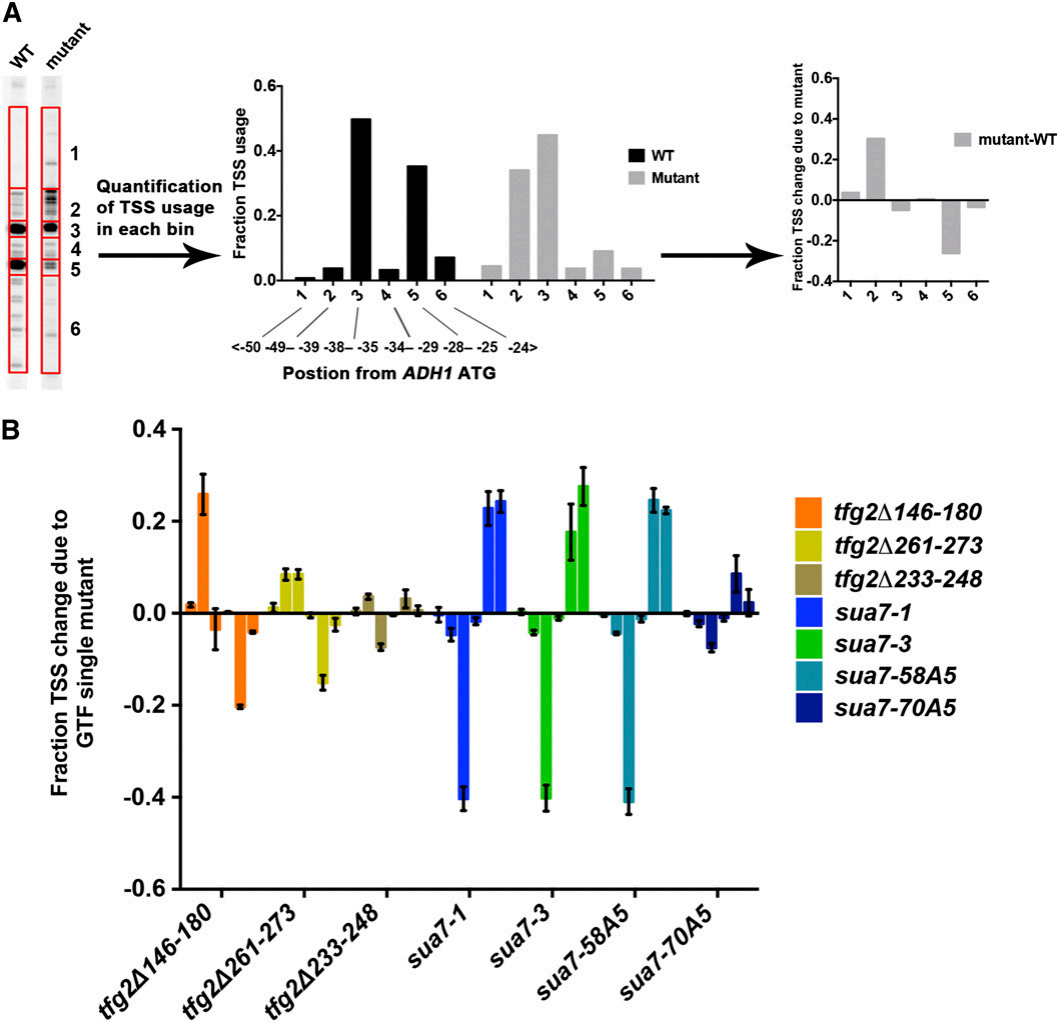
Transcription start site (TSS) usage distribution at *ADH1* and its alteration by Pol II GTF mutants. (A) TSSs detected by primer extension at *ADH1* are distributed over a range of positions. To quantify TSS distributions, *ADH1* start site signals were divided into six bins separated by promoter position and normalized to total signal for each lane (left panel). TSS usage distributions were quantified for different strains (middle panel). Relative change in normalized TSS usage distribution for mutant compared to WT (negative numbers indicate relative decrease in TSS position usage; positive numbers indicate relative increase) is then calculated and plotted (right panel). (B) Alterations in TSS usage at *ADH1* caused by each GTF mutant shown were quantified as in (A). Start site defects of these GTF mutants are consistent with previous publications (Wu *et al.* 1999; Eichner *et al.* 2010), except for *sua7-A5* alleles, which are in contrast to (Zhang *et al.* 2002). Graphs show average of at least three independent determinations with error bars representing SDs. See Figure S1 for representative primer extension experiments.

We used changes in model gene *ADH1* TSS distribution as a proxy for *in vivo* initiation defects. To quantify changes in TSS distribution at *ADH1*, signals from *ADH1* TSSs were placed into six bins and alterations in the fraction of TSSs present in each bin were determined relative to the WT distribution (Figure 1A). We first examined how GTF mutants—known to alter TSS on their own—altered TSS defects of Pol II mutants and whether they modulated Pol II mutant growth phenotypes to explore their possible influence on TSS defects of Pol II mutants and characterized any effects in light of any genetic interactions between Pol II mutants and GTF alleles. We wished to determine if opposite shifting Pol II and GTF TSS mutants were suppressive or additive when combined, for example, similarly to the combination of TFIIB and TFIIF alleles or combination of Pol II GOF and LOF mutants exhibiting suppression of TSS defects and growth phenotypes. Conversely, we might observe nonadditive behavior in double mutants, indicative of bypass or epistasis as we observed between *sub1∆* and Pol II GOF alleles (Braberg *et al.* 2013). If a double mutant has a defect in growth phenotype that is better than expected from examination of individual phenotypes of single mutants [based on a multiplicative model for double mutant growth interactions (Schuldiner *et al.* 2006)] or an additive model for TSS defects, such an observation can be an example of epistasis. In such cases, single mutants would show a lack of independence when combined, with double mutants exhibiting phenotypes of one or the other single mutant, or a phenotype worse than either single mutant but to a lesser degree than would be expected from independently acting mutations. Finally, we asked whether GTF-Pol II genetic interactions and growth phenotypes strictly correlated with any observed modulation of Pol II mutant TSS defects.

We integrated three deletion mutants of *TFG2* into a yeast strain designed for phenotyping *rpo21/rpb1* alleles. *tfg2∆146-180* and *tfg2∆261-273* had been shown to shift *ADH1* TSSs upstream, whereas *tfg2∆233-248* had been shown to exhibit a mild *ADH1* TSS phenotype at best (Eichner *et al.* 2010). We also utilized *SUA7* alleles containing substitutions in the “B-reader region”: *sua7-1* (encodes E62K in TFIIB), *sua7-3* (encodes R78C in TFIIB) alleles that have been shown to confer downstream TSS shifts by the Hampsey group (Pinto *et al.* 1994; Wu *et al.* 1999), and *sua7-58A5* and *sua7-70A5* mutants previously described as having upstream shift effects (Zhang *et al.* 2002). *sua7-58A5* and *sua7-70A5* each contain an insertion of five alanines at different positions in the B-reader (amino acid 58 or 70, respectively) and were recreated in our laboratory based on the published description by Zhang *et al.* (2002). Effects of GTF single mutants on *ADH1* TSS distribution are shown in Figure 1B. *tfg2∆146-180* has stronger upstream shifts at *ADH1* than *tfg2∆261-273*, whereas *tfg2∆233-248* has little effect, consistent with the work of Eichner *et al.* (2010). *sua7-1* and *sua7-3* show strong downstream shifts as shown previously (Pinto *et al.* 1994; Wu *et al.* 1999); however, *sua7-58A5* and *sua7-70A5* both show downstream shifts, strong and weak, respectively, which is typical behavior of *sua7* TSS mutants but is opposite of published observations. We cannot explain this discrepancy but consider *sua7-58A5* and *sua7-70A5* to have standard behavior for *sua7* alleles.

**Figure 2:**
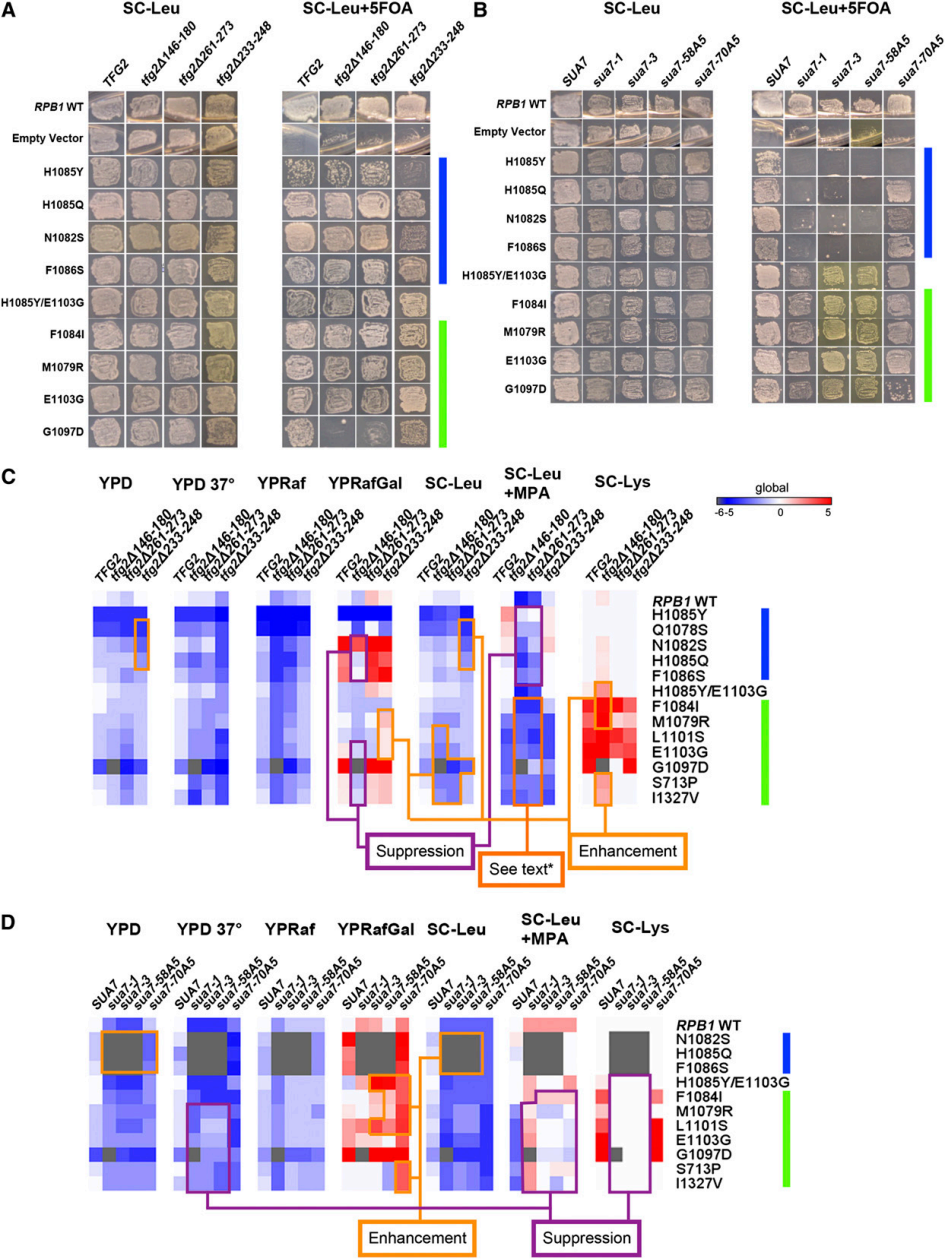
Genetic interactions between GTF and Pol II mutants. (A) Plasmids containing *tfg2* mutants were integrated into yeast strains constructed to allow shuffling of a WT *RPO21/RPB1 URA3* plasmid in favor of WT or mutant *rpo21*/*rpb1 LEU2* plasmids through use of 5FOA poisoning of *URA3*^+^ cells. On the left, SC-Leu media allows coexistence of both *URA3* and *LEU2* plasmids. On the right, supplementation of SC-Leu with 5FOA uncovers *rpb1* phenotypes by selecting against the *RPB1 URA3* plasmid. Pol II mutants that have slower elongation rate than WT *in vitro* (LOFs) and mutants that genetically cluster with them are annotated with a blue bar; mutants that have faster elongation rate than WT *in vitro* (GOFs) and mutants genetically cluster with them are labeled with a green bar. Mutants are arranged by their measured elongation rates or elongation rates inferred by strength of genetic phenotypes compared with those mutants tested biochemically (Kaplan *et al.* 2012). (B) *sua7* allele-Pol II mutant interactions examined as for *tfg2* alleles in (A). (C and D) Phenotypes of viable GTF-Pol II double mutants are shown as a heatmap with qualitative determinations of growth defects on various media. Inviable double mutants are colored in gray. In YPD, YPD 37°C, YPRaf, and SC-Leu media, single and double mutant growth levels are normalized to WT. Blue indicates decreased growth relative to WT; red indicates increased growth compared with WT. In SC-Leu+MPA, growth difference is normalized to that on SC-Leu to quantify MPA sensitivity (shown as blue) or resistance (shown as red). In YPRafGal and SC-Lys, growth on the plate is divided by ratio of WT growth to mutant growth on corresponding general media (YPD and SC-Leu) to account for Gal^R^ and Spt^-^ phenotypes (Gal^+^ and Lys^+^, shown in red) in contrast to their underlying growth defects. (C) *tfg2* mutants. (D) *sua7* mutants. See Figure S2 and Figure S3 for representative spot growth assay figures used for quantification in heatmaps and see *Materials and Methods* for further explanation of heatmaps.

We tested the growth of these GTF mutants alone or in combination with Pol II mutants by transformation and plasmid shuffling of *rpo21/rpb1* alleles in place of *RPO21*. Genetic interactions between GTF alleles and Pol II mutants appear complex, and we first describe genetic interactions apparent on growth of strains in rich or defined media (Figure 2, A and B; YPD and SC-Leu media described in Figure 2, C and D), followed by observed genetic interactions relating to gene-specific transcriptional phenotypes (Spt^−^, Gal^R^, MPA sensitivity) (Figure 2, C and D).

When GTF alleles and Pol II mutants were combined, we observed allele-specific interactions between GTF alleles and Pol II GOF/LOF mutant classes. When Pol II GOF mutants and *tfg2* alleles were combined, the most severe GOF mutant *rpo21/rpb1* G1097D exhibited a strong negative interaction with *tfg2∆146-180* or *tfg2∆261-273* (lethality and synthetic sickness, respectively). LOF Pol II mutants (downstream TSS shifting mutants) and *tfg2∆146-180* and *tfg2∆261-273* (upstream TSS shifting mutants) did not result in suppression of growth defects of Pol II LOF mutants on standard rich or defined media in contrast to mutual suppression of growth defects when Pol II GOF and LOF (opposite TSS shifting mutants) are combined (Kaplan *et al.* 2012), or when *tfg* and *sua7* alleles (opposite TSS shifting mutants) have been combined (Sun and Hampsey 1995; Ghazy *et al.* 2004; Freire-Picos *et al.* 2005) (Figure 2, A and C). The *tfg2∆233-248* allele, which does not have a clear TSS defect, exhibited a negative interaction with Pol II LOF alleles but no clear interactions with Pol II GOF alleles. Neither *tfg2* upstream shifting allele could rescue lethal LOF Pol II mutants (Figure S2A), in contrast to rescue of lethal Pol II LOFs when combined with GOF mutants (Kaplan *et al.* 2012).

When *sua7* mutants were combined with GOF Pol II mutants (upstream TSS shifting mutants), we observed apparent lack of additivity in growth defects of strong downstream shifting *sua7* alleles combined with Pol II GOFs. The double mutants generally showed growth defects in between those of the single mutants, suggesting directional suppression or epistasis, but not mutual suppression, where double mutants would be expected to grow better than either single mutant (mutual suppression is generally observed when Pol II LOF and GOF trigger loop mutants combined within the same enzyme). These effects were more pronounced on YPD medium than on YPRaf or defined medium (SC-Leu). We also observed partial suppression of the sensitivity to increased temperature (37°, Ts^−^ phenotype) of *sua7-1*, *sua7-3*, and *sua7-58A5* alleles by Pol II GOF alleles (Figure 2, Figure S3). The weak downstream shifting allele *sua7-70A5* enhanced growth defects with Pol II GOF alleles, distinct from the other stronger *sua7* alleles tested.

In contrast to the milder phenotypes of *sua7*-Pol II GOF strains, Pol II LOF mutants were exquisitely sensitive to defects in TFIIB, as widespread synthetic lethality or sickness was observed between *sua7* alleles and Pol II LOF mutants. *sua7-1*, *sua7-3*, and *sua7-58A5* showed very strong negative interactions with all LOF Pol II mutants tested, with most double mutants being inviable. A weak downstream shifting allele, *sua7-70A5*, showed negative but weaker interactions with Pol II LOF alleles. Both classes of mutant, Pol II LOF and *sua7* alleles, alter TSS distributions in a similar fashion, suggesting exacerbated TSS defects might underlie observed synthetic genetic interactions (see below). These results indicate that combinations of Pol II and GTF mutants exhibiting the same polarity of TSS defects can lead to exacerbation of growth defects, wherein aggravated initiation defects may be a major contributor of the observed growth defects. The strongest genetic interaction between Pol II mutants and GTF alleles was observed when Pol II LOF mutants and strong *sua7* downstream shifting alleles were combined (lethality), suggesting that Pol II LOF mutants are much more sensitive to initiation defects than GOF mutants. Combinations of mutants with opposing TSS distribution defects resulted in partial, but not necessarily mutual, suppression of single mutant growth defects on generic media.

We also examined conditional growth phenotypes on several other types of media, including those reporting on gene-specific transcription defects *in vivo* (Kaplan *et al.* 2012; Braberg *et al.* 2013). Results are shown as a heatmap of normalized estimates of phenotypic strength as determined by visual determination of growth on plates (*tfg2* upstream shifting alleles in Figure 2C, *sua7* downstream shifting alleles in Figure 2D; see Figure S2 and Figure S3 for representative images; see *Materials and Methods* for calculations). We observed sensitivity to mycophenolic acid (MPA) for *tfg2* alleles, likely corresponding to upstream TSS shifts causing inability to induce *IMD2* in the presence of MPA, with *tfg2∆146-180* the most MPA-sensitive. MPA sensitivity has been shown to correlate well with upstream TSS shifts of a subset of Pol II mutants, including those tested here (Braberg *et al.* 2013). Conversely, *sua7* alleles appeared resistant to MPA, similar to Pol II LOF alleles (Figure 2, C and D). When Pol II GOF alleles are combined with the strong upstream shifting allele *tfg2∆146-180*, the double mutants are inviable on this medium. The weaker upstream shifting allele *tfg2∆261-273* also exacerbates MPA sensitivity of Pol II GOF alleles, with the resulting double mutants exhibiting no growth on this medium. This enhancement of MPA sensitivity leading to double mutants’ lack of detectable growth is not well-illustrated in our heatmap due to our calculation metric being unable to capture zero growth during calculation of “net” MPA sensitivity (see *Materials and Methods*). In contrast, MPA sensitivities of *tfg2* alleles were suppressed when combined with Pol II LOFs; similarly, MPA sensitivities of Pol II GOFs were suppressed when combined with all *sua7* alleles, being more apparent in stronger *sua7* downstream shifting alleles. Therefore, MPA phenotypes of combinations of GTF alleles and Pol II mutants appeared additive or suppressive depending on the nature of the allele class: when upstream shifting alleles were combined, MPA sensitivity was exacerbated; when an upstream shifting mutant was combined with a downstream shifting mutant, MPA sensitivity was alleviated (Figure 2, C and D). These results support MPA sensitivity as a readout for initiation defects and not necessarily elongation defects, as widely assumed, and predict that TSS defects of Pol II mutants and GTF alleles may be additive and suppressive. *sua7* alleles with strong TSS defects showed strong temperature sensitivity (Ts^−^); however, this could partially be alleviated when combined with Pol II GOF mutants (Figure 2D).

We also examined gene-specific transcription-related Gal^R^ and Spt^−^ phenotypes, which have less clear relationships to TSS defects (see *Materials and Methods*). Most Pol II GOF mutants do show Spt^−^ phenotype as measured by suppression of lysine auxotrophy (Lys^−^) in the presence of *lys2-128∂* allele (see *Materials and Methods*), but there is only partial correlation with upstream shifting TSS defects. *tfg2∆146-180* enhanced Spt^−^ phenotypes of all moderate Pol II alleles, whereas the other *tfg2* alleles did not show apparent interaction with Pol II alleles for the Spt^−^ phenotype. Stronger downstream shifting *sua7* alleles suppressed Spt^−^ phenotypes of Pol II GOF alleles (Figure 2, C and D). A number Pol II LOF and GOF mutants have been shown to exhibit the Gal^R^ phenotype in the presence of the *gal10∆56* allele of *GAL10* (see *Materials and Methods* for description) (Kaplan *et al.* 2012). The strongest upstream shifting allele *tfg2∆146-180* suppressed Gal^R^ phenotypes of all Pol II alleles that had the Gal^R^ phenotype, whether GOFs or LOFs; *tfg2∆233-248*, an allele with no apparent *ADH1* TSS defect, enhanced Gal^R^ phenotypes of weak Pol II GOF alleles. *sua7* alleles showed Gal^R^ phenotypes on their own and enhanced those of Pol II GOF alleles (Figure 2, C and D). The wide range of genetic interactions including enhancement and suppression of these conditional growth phenotypes suggests a complex network between Pol II and TFIIB/TFIIF that may relate to gene-specific effects not apparent in overall double mutant growth phenotypes.

### Combinations of GTF alleles and Pol II alleles lead to mutual suppression of TSS defects but not mutual suppression of generic growth defects

Because we observed above that GTF TSS defective mutants and Pol II TSS defective mutants conferred enhanced growth defects when same-direction TSS shifting mutants were combined but showed mostly weak, conditional, or directional suppression when opposite direction shifting mutants were combined, we examined how each class of double mutant affected TSS distribution at *ADH1*. This was performed to determine if TSS defects of combination of GTF alleles and Pol II mutants were additive and suppressive similar to MPA sensitivity phenotypes or were exacerbating, but not mutually suppressive, similar to general growth phenotypes of double mutants.

**Figure 3:**
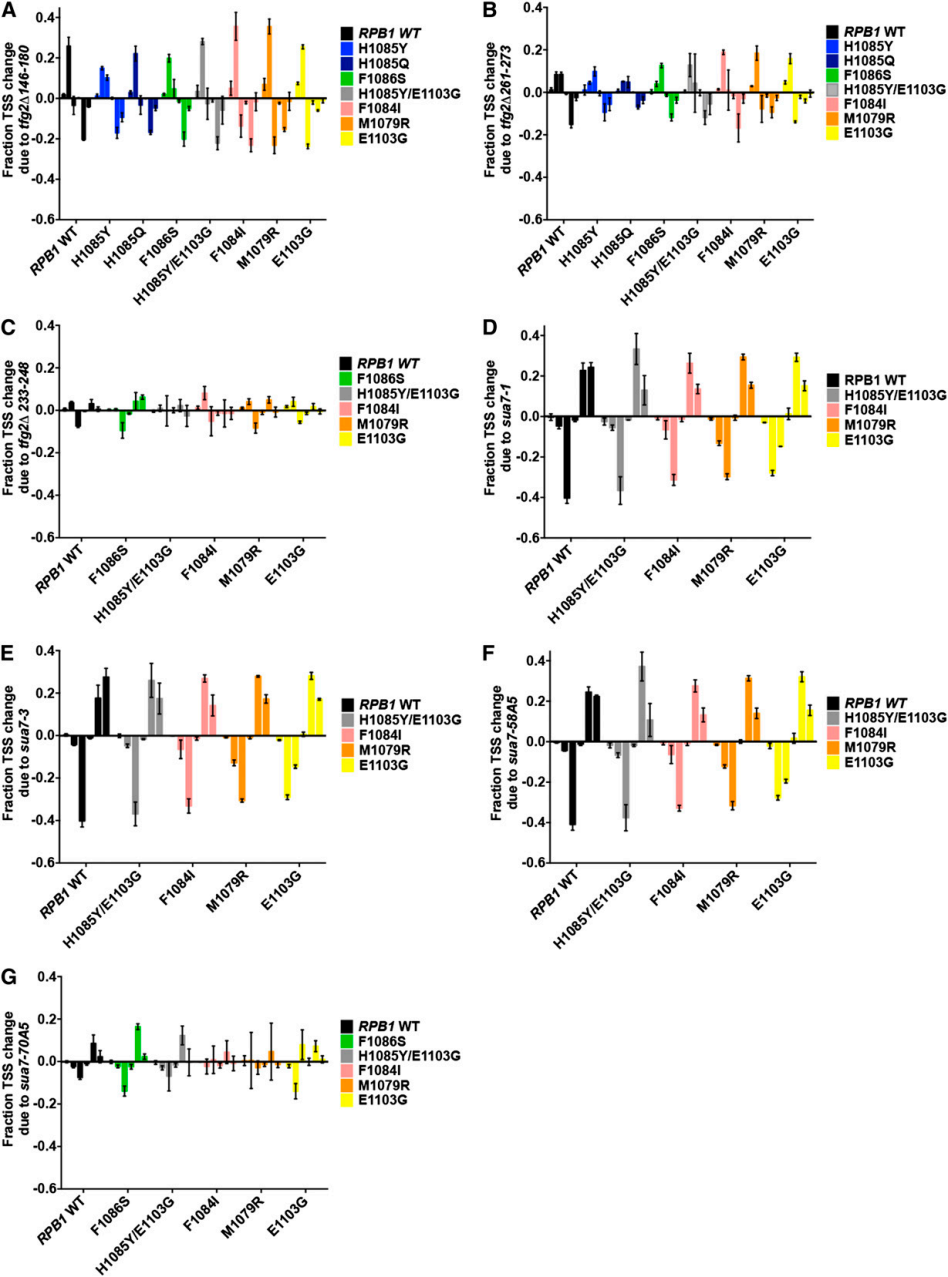
Modulation of Pol II mutant TSS selection defects at *ADH1* by GTF mutants. (A) Quantification of effects of GTF alleles on Pol II alleles on TSS utilization by comparison of double mutants to respective Pol II single mutants at *ADH1* (quantified as in Figure 1A). Values indicate average of a minimum of three independent determinations, with SDs represented by error bars. (A.) *tfg2∆146-180*. (B) *tfg2∆261-273*. (C) *tfg2∆233-248*. (D) *sua7-1*. (E) *sua7-3*. (F) *sua7-58A5*. (G) *sua7-70A5*. See Figure S1 for representative primer extension experiments.

We observed that the TSS defects of GTF mutants and Pol II mutants were uniformly additive or suppressive, depending on the direction of TSS shifts of individual mutants, but not epistatic (Figure 3, see Figure S1 for representative raw data). *tfg2∆146-180* and *tfg2∆261-273* mutants shifted *ADH1* TSSs upstream relative to all single Pol II alleles tested, indicating exacerbation of Pol II GOF alleles that shift *ADH1* TSSs upstream on their own and suppression of Pol II LOF alleles that shift TSSs downstream on their own (Figure 3, A and B). The mutual suppression of TSS defects observed was similar to those observed for double mutant combinations of TFIIF and TFIIB alleles or for intra-Pol II double mutants. Conversely, those cases were accompanied by mutual suppression of growth phenotypes, which appears lacking for combinations of GTF alleles with Pol II trigger loop alleles (Figure 2). Additionally, strong downstream shifting alleles *sua7-1*, *sua7-3*, *sua7-58A5* shifted *ADH1* TSSs of *sua7*-Pol II GOF double mutants downstream relative to all Pol II GOF single mutants (Figure 3, D–F), indicating additive effects of opposite polarity shifts and, therefore, suppression of GOF Pol II allele TSS defects at *ADH1*. *sua7-70A5*, a weak downstream shifting allele, shifted *ADH1* TSSs of *sua7-70A5*-Pol II double mutants downstream relative to all Pol II allele backgrounds (Figure 3G), indicating exacerbated downstream TSS shifts of LOF Pol II alleles and suppression of upstream TSS shifts of GOF Pol II alleles. TSS defects and generic and conditional growth phenotypes tested in GTF-Pol II double mutants suggest that TSS defects may contribute to general growth defects when TSS defects are severe, yet suppression of TSS defects (as measured at *ADH1*) does not correlate with suppression of Pol II mutant generic growth defects. Taken together, these results suggest that TSS defects may contribute to Pol II mutant growth defects, and Pol II activity alterations can partially compensate for defects in GTFs, but initiation defects are not likely to be the main or only drivers of observed Pol II allele growth phenotypes.

### Genetic interactors with widespread genetic interactions with Pol II TSS defective alleles do not generally have TSS defects on their own or when combined with Pol II alleles

**Figure 4:**
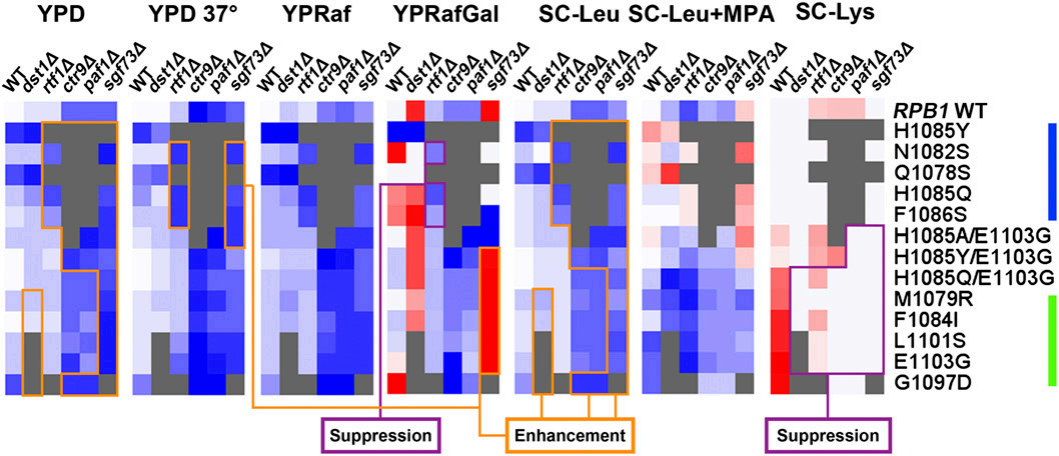
Genetic interactions between Pol II alleles and genetic interactor deletions. Phenotypes of genetic interactor deletions on their own or in combination with Pol II mutants on different medium normalized to WT are shown in the heatmap. See description in Figure 2, C and D and see *Materials and Methods* for heatmap details. See Figure S7B in Braberg *et al.* (2013) and Figure S5 for representative spot growth assays used for heatmaps.

**Figure 5:**
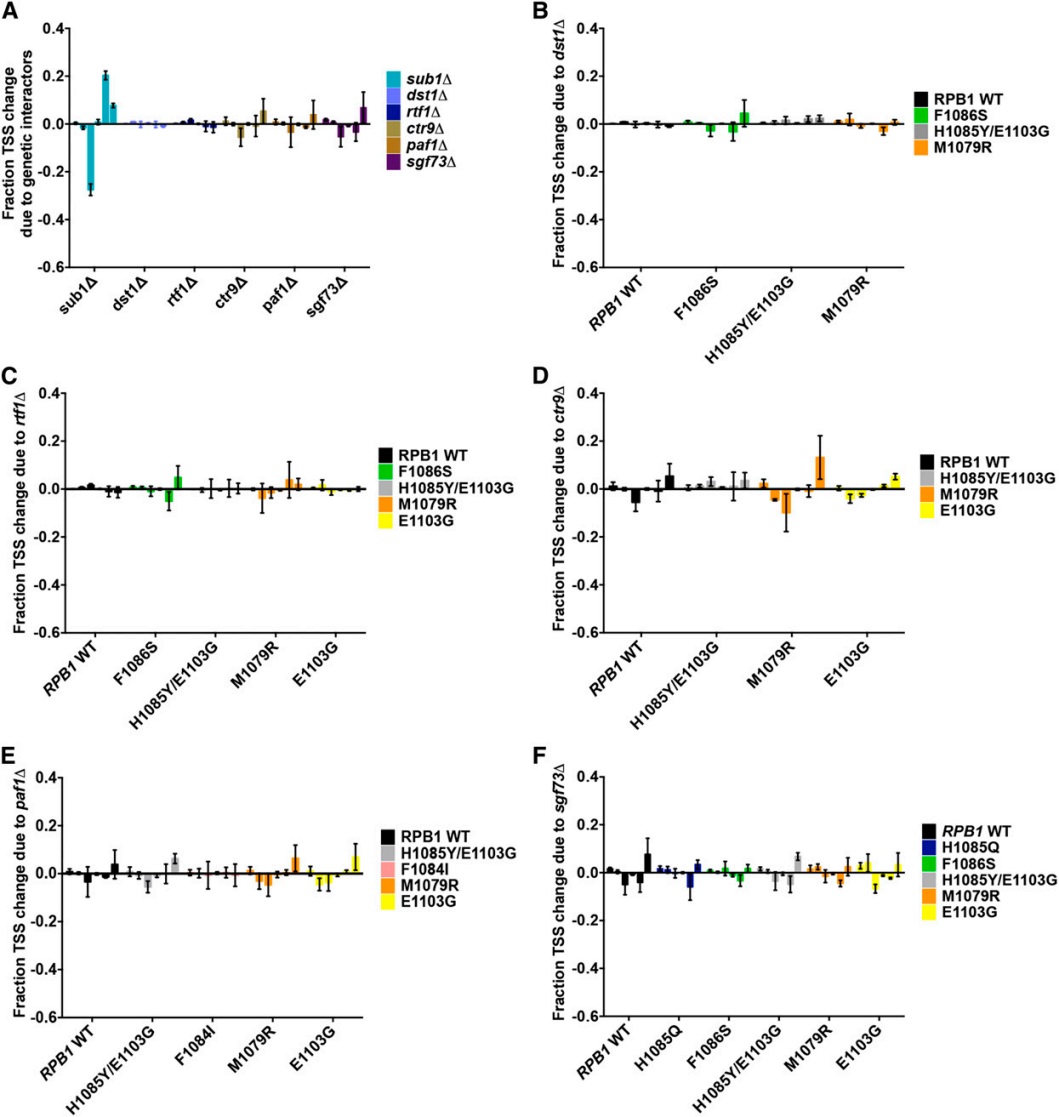
Genetic interactors do not generally modulate TSS defects of Pol II mutants at *ADH1*. (A) Quantification of TSSs usage alterations relative to WT at *ADH1* in genetic interactor deletions are shown (quantified as in Figure 1A). Values indicate a minimum of three independent determinations, with SDs represented by error bars. (B–F) Quantification of TSSs usage distribution differences between genetic interactor deletion—Pol II double mutants relative to Pol II single mutants are shown. (B) *dst1∆*. (C) *rtf1∆*. (D) *ctr9∆*. (E) *paf1∆*. (F) *sgf73∆*, respectively. See Figure S4 for representative primer extension experiments.

Inspired by the discovery of a Pol II genetic interactor, *sub1∆*, that conferred a downstream TSS defect on its own and enhanced downstream TSS defects and growth defects of LOF Pol II alleles but showed epistasis with GOF Pol II alleles for TSS defects and growth phenotypes (Braberg *et al.* 2013), we investigated how factors that have genetic interactions with Pol II TSS defective alleles affected TSS distributions on their own or in combination with Pol II alleles. Are genetic interactions of factors and Pol II TSS shifting alleles predictive of their effects on TSS utilization? The genetic interactions of *dst1∆*, *rtf1∆*, *sgf73∆*, *paf1∆*, *and ctr9∆* with Pol II alleles have been shown previously (Hartzog *et al.* 1998; Malagon *et al.* 2006; Braberg *et al.* 2013). These and additional factors are illustrated in Figure 4. Deletion of *DST1* (encoding the general transcription elongation factor TFIIS) showed allele-specific genetic interactions with GOF Pol II alleles and suppressed the Spt^−^ phenotypes of Pol II alleles. *RTF1*, *CTR9*, *PAF1* (genes encoding subunits of the Paf1C complex) showed stronger genetic interactions with Pol II LOF alleles and exhibited mild Spt^−^ phenotypes on their own but suppressed the Spt^−^ phenotypes of Pol II alleles. However, *SGF73* (encoding a subunit of the histone-modifying SAGA complex) showed genetic interactions with both classes of alleles, enhanced MPA resistance of Pol II LOF alleles, and suppressed Spt^−^ phenotypes of Pol II alleles (Figure 4). We found that deletions of these genetic interactors did not confer any strong TSS defects at *ADH1* on their own (Figure 5A), nor did they modulate TSS defects of either LOF or GOF Pol II alleles (Figure 5B). The genetic interactions these factors exhibit with Pol II TSS-defective alleles may go through distinct mechanisms from *sub1∆*, whose genetic interactions on growth mirrored its effects on TSS defects at *ADH1* when combined with Pol II alleles (Braberg *et al.* 2013). These results suggest genetic interactions between factors and Pol II alleles are not predictive of initiation defects, and that growth defects of many or most double mutant combinations with Pol II alleles do not result from exacerbation of TSS defects, under the assumption that *ADH1* is a proxy for global TSS defects (supported by our unpublished global analysis of TSS defects in Pol II mutants).

## Discussion

Combination of mutant alleles allows the relationships of different factors to be probed, with the hope of revealing distinctions between their contributions to different processes and possibly suggesting mechanism. A number of factors contribute to TSS selection in *S. cerevisiae* and their relationships have been probed here to understand the requirements for normal Pol II initiation. Previous analyses showing that distinct classes of TSS mutant exist in yeast (upstream and downstream shifting), supporting a scanning model for identification of TSSs. When mutants of differing TSS shift class have been combined (Sun and Hampsey 1995; Ghazy *et al.* 2004; Freire-Picos *et al.* 2005; Kaplan *et al.* 2012), they generally exhibited mutual suppression of TSS defects coupled to suppression of growth defects, whereas mutants of the same class exhibited enhancement of TSS and growth defects.

We previously discovered that *SUB1* (homolog of PC4) has wide-ranging genetic interactions with Pol II mutants in a manner correlating with class of Pol II mutant (GOF or LOF) (Braberg *et al.* 2013). *SUB1* was originally genetically isolated as a high copy suppressor of TFIIB alleles and biochemically as a positive transcription factor that stimulates basal transcription (Henry *et al.* 1996; Knaus *et al.* 1996). Deletion of *SUB1* causes synthetic lethality in combination with *sua7* TSS defective mutants (Knaus *et al.* 1996). The strong genetic interactions between *SUB1* and *SUA7* suggested a close association of their function in initiation and TSS selection and *in vivo* growth. We found that *sub1∆* caused *ADH1* TSS distribution to shift downstream and exacerbated the downstream shifts of Pol II LOF mutants, correlating with exacerbation of growth defects in *sub1∆*-Pol II LOF mutant double mutants (Braberg *et al*., 2013). Distinct from the general trend conferred by combination of TSS shifting alleles mentioned above, *sub1∆* TSS effects were not additive with Pol II GOF mutants; instead, epistasis was observed for Pol II GOF alleles combined with *sub1∆* (Braberg *et al.* 2013). This epistasis was in contrast to *sub1∆* enhancement of Pol II LOF alleles for both growth phenotypes and TSS shifts. In light of these different classes of relationships previously observed among mutants altering TSS selection, we examined the relationships between GTF alleles and Pol II alleles with altered trigger loops and relationships between these same Pol II TL alleles and other Pol II genetic interactors.

**Figure 6:**
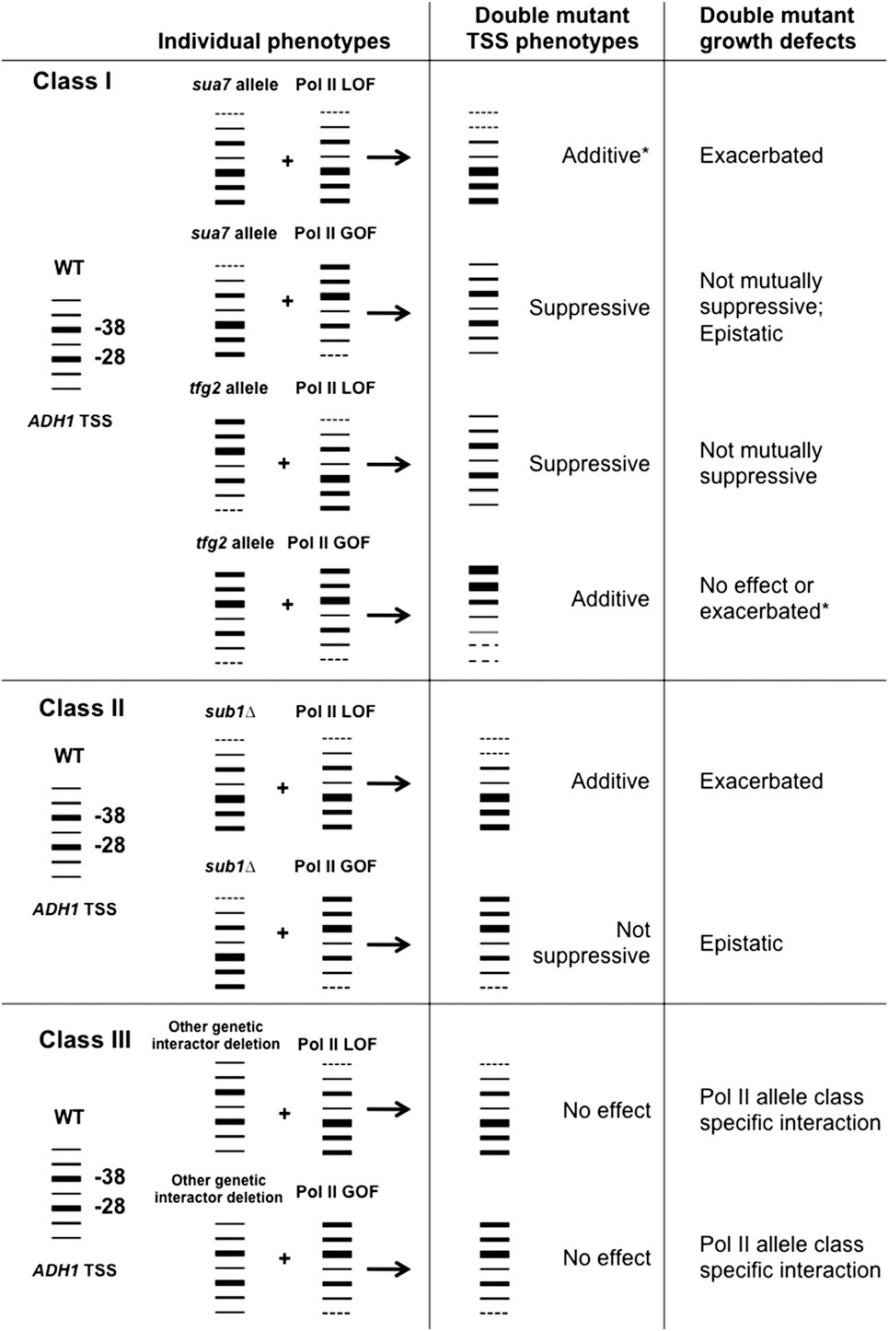
Model of relationships between TSS distribution shifting GTF alleles, Pol II active site alleles, and other genetic interactors. GTF alleles and Pol II mutants have additive or suppressive effects on TSSs at *ADH1* while showing exacerbation when same direction TSS mutants are combined, but no mutual suppression of growth defects when opposite polarity TSS mutants are combined (Class I); *sub1∆* has enhancement or epistasis with Pol II alleles on both TSS defects and growth defects, which is thus far unique among tested mutants (Class II), whereas other tested genetic interactors do not modulate TSS defects on their own or in combination with Pol II alleles but exhibit a wide range of genetic interactions with Pol II TSS shifting mutants, suggesting relationships based on defects outside of TSS selection or initiation (Class III). *Description based on viable double mutants.

We found that, unlike previous combinations of TSS shifting alleles, suppression of TSS defects by Pol II mutant-GTF combinations could be partially separated from their effects on growth. Furthermore, we found that previous observations regarding the relationship of *sub1∆* with Pol II alleles for TSS determination were relatively unique compared with a number of other Pol II genetic interactors examined here. Taken together, we can now discern at least three types of genetic relationships among TSS-altering alleles, Pol II interacting alleles, and Pol II active site mutants in regard to double mutant modulation of TSS and growth defects (Figure 6). First, GTF and Pol II mutants, each altering start sites on their own, have additive or suppressive effects on TSS distribution at *ADH1*, depending on the nature of their single mutant defects (Class I). Unlike combinations of GTF alleles and a subset of previously examined *rpb* alleles, TSS defect suppression is partially uncoupled from growth defect suppression for oppositely acting TSS alleles. Negative growth interactions between GTF alleles and Pol II alleles correlated with exacerbated TSS defects but were of much greater strength for downstream shifting GTF TSS alleles and Pol II LOF alleles than for upstream shifting GTF TSS alleles and Pol II GOF alleles. These observations suggest that *S. cerevisiae* growth is much more sensitive to defects in initiation that result from decreased initiation efficiency. Second, *sub1∆* is thus far unique in the strong epistasis observed between Pol II GOF alleles and *sub1∆* for TSS selection at *ADH1* (Class II). This indicates that *sub1∆* defects are distinct from those of TFIIB alleles, and that while *sub1∆* appears to be bypassed in Pol II GOF alleles, *sua7* defects are not, although there is some observable suppression of specific phenotypes in *sua7*-Pol II GOF mutant strains. Third, other tested genetic interactors with Pol II do not generally have TSS defects on their own or modulate Pol II TL mutant TSS defects (Class III), suggesting that the strong correlation between TSS defects and a very broadly Pol II interacting mutant, *sub1∆*, is relatively unique. Genetic interactions we observe between these factors and Pol II mutants may be originated (caused) by other transcriptional defects.

Although our experiments suggest that Pol II activity–dependent growth defects can be uncoupled from observed TSS defects, open questions remain concerning the mechanisms by which Pol II start sites are determined. RNA polymerases prefer to initiate at YR (−1, +1) sites, where the initiating nucleotide of an RNA is a purine, with a pyrimidine just upstream. A crystal structure of a viral RNA polymerase suggested that this sequence preference was likely due to purine stacking between the initiating NTP and a purine at the −1 position on the template strand (meaning a pyrimidine at −1 on the transcribed strand), and a set of very recent bacterial RNAP structures confirm this for multisubunit RNAPs (Gleghorn *et al.* 2011; Basu *et al.* 2014; Zhang *et al.* 2014). It seems likely that there are additional sequence determinants controlling efficiency of usage of any particular start site. For example, at *ADH1* transcription mutants appear to alter the probability of usage of YR sequences that are used by WT present in the start region. Although Pol II GTFs might be positioned within the Pol II PIC to interact with sequences and “read” for the start site, the primary determinants for TSS preference are the −1/+1 bases that are located deep in the active site and not bases juxtaposed to hypothetical or predicted GTF locations in the PIC (these would mostly be upstream sequences). Moreover, the types of TSS changes observed for GTF mutants are phenocopied by mutations in the Pol II trigger loop, suggesting they might arise from similar types of defects in transcription. What could these similar defects be?

The altered patterns of starts observed in most upstream or downstream shifting start site mutants appear to be stereotypical to each class, meaning the positions of starts that are more likely used at *ADH1* in mutants are the same within each mutant class as defined by upstream or downstream shifting, not by whether they are in GTF subunits or the Pol II active site. However, the fraction of usage on these usable start sites differs (strong or weak shift in distribution of TSS usage) depending on how much an allele deviates from WT catalytic activity for Pol II alleles or on how strong they appear genetically (for GTF alleles). In other words, different mutants may not necessarily alter initiation sequence *preference*, but instead may shift the initiation *probability* of use in a polar fashion within some set of already usable start sites. In this view, Pol II initiation efficiency may cooperate with a directional scanning process that has its own rate. Pol II with increased catalytic activity enables initiation earlier within the scanning window, increasing catalytic efficiency (initiation probability) of earlier usable start sites usages, and shifting TSS usage distribution upstream.

Additive effects on *ADH1* TSS distributions in Pol II-GTF mutant strains indicate that individual defects of each allele are present in the double mutant strains. How might we understand these defects? Defects in *sua7* are consistent with defects in initiation efficiency and likely represent reduced TFIIB functions. TFIIB function in concert with the Pol II active site might represent communication between TFIIB and the active site as has been proposed (Sainsbury *et al.* 2013) or parallel roles for TFIIB and the Pol II active center during putative TSS scanning. We speculate that *sua7* Pol II LOF double mutants that have severe growth defects or are lethal as shown in Figure 2 have exacerbated defects in initiation efficiency from those of single mutants, *e.g.*, those shown for *sua7-1* (Cho and Buratowski 1999). Furthermore, mutual suppression of TSS defects in GTF-Pol II double mutants is predicted to result from suppression of initiation defects, which might be tested for GTF-Pol II mutant combinations in GTF-dependent biochemical systems for abortive or productive Pol II initiation such as the system used recently by Fishburn and Hahn (2012). Because Pol II alleles are expected to have additional defects in elongation and termination, GTF alleles do not strongly suppress overall growth defects of Pol II mutants even though TSS defects in some cases examined here are strongly suppressed. Our previous work detecting splicing defects as a consequence of Pol II alleles’ presumed altered elongation functions did not detect similar splicing defects for GTF alleles. Defects detected were milder and of opposite polarity for *sua7-3* and *tfg2∆261-273* strains relative to Pol II alleles with upstream or downstream shifts in TSS distributions, arguing against predictable defects of these alleles in Pol II elongation (Braberg *et al.* 2013).

*tfg2* TSS phenotypes are similar to those of Pol II GOF alleles and raise the question of how alteration of TFIIF function alters TSS distribution. Previous work had indicated that a *TFG1* mutant exhibited an increased ability to stimulate Pol II activity in an abortive initiation assay (Khaperskyy *et al.* 2008). Fishburn and Hahn (2012) recently reported a negative role for TFIIF in suppressing TSS usage at upstream positions of promoters (nearer to a TATA element). Such a negative role is likely balanced by positive requirements for TFIIF activity in promoting initiation. Conceivably, *tfg1* and *tfg2* mutants have this negative role—possibly an autoinhibitory function of TFIIF that is alleviated during scanning to downstream positions—specifically or selectively compromised. In light of such a model, upstream TSS shifting *tfg* alleles would confer increased initiation activity, phenocopying Pol II GOF mutants for altered TSS distribution. In *in vitro* transcription experiments, TFIIF stimulation of abortive initiation was compromised by the Pol II LOF H1085Y allele of the TL (Cabart *et al.* 2014). TFIIF stimulation of abortive initiation likely represents one of a number of positive TFIIF roles in initiation. If the observed *in vitro* stimulation were related to upstream TSS shifts at *ADH1* observed in *tfg* alleles, then compromise of the TL might be expected to be epistatic to *tfg* phenotypes based on biochemical results. Because *tfg2* alleles can still alter TSS distribution in Pol II LOF alleles such as H1085Y, it suggests that the biochemical requirement *in vitro* of TFIIF for a WT TL may be distinct or bypassed by *tfg2* alleles studied here.

The mechanism driving *S. cerevisiae* start site scanning likely derives from a combination of factors, Pol II activity and transcription bubble opening promoted by TFIIH. Assuming that upstream bubble opening, as was observed at *GAL1* and *GAL10* (Giardina and Lis 1993), is universal at yeast promoters, a major question is, how does the bubble transit to the distal start sites? One possibility is that a large transcription bubble is extended to the start site region. In this case, PICs would need to accommodate extensive single-stranded DNA. A recent cryo-EM structure and model of the yeast PIC appears consistent with such accommodation (Murakami *et al.* 2013). In this instance, TFIIH might drive extension of the downstream bubble edge through the activity of its Ssl2/Rad25 subunit (the yeast homolog of human ERCC3/XPB). An alternative model in which a smaller region of melted DNA translocates along with the open PIC toward the start region may also be possible, but almost nothing is known of the organization of nucleic acids in such hypothetical complexes or how translocation of the putative bubble would be controlled during initiation. In translocating Pol II elongation complexes, GTFs are not present and interactions between Pol II, both DNA strands, and nascent RNA organize the upstream edge of the transcription bubble.

Genetic analyses have allowed us to examine the relationships between Pol II activity mutants, known initiation factors, and candidates for possible modifiers of Pol II initiation activity. Our experiments indicate that Pol II genetic interactors need not perturb TSS selection, and that initiation defects are likely only a partial driver of Pol II allele growth phenotypes. Altered Pol II activity through TL defects do not bypass or appear epistatic to the alleles of TFIIB or TFIIF studied here for *ADH1* TSS selection, suggesting that they each function separately as part of a concerted process to promote efficient TSS selection.

## Supplementary Material

Supporting Information
