# Supplementary material for: Relationships of RNA Polymerase II Genetic Interactors to Transcription Start Site Usage Defects and Growth in Saccharomyces cerevisiae
Source: G3 (Bethesda). 2014 Nov 6;5(1):21–33. doi: 10.1534/g3.114.015180 (PMC4291466; doi:10.1534/g3.114.015180)
Supplement: Supporting Information [file supp_5_1_21__index.html]

Relationships of RNA Polymerase II Genetic Interactors to Transcription Start Site Usage Defects and Growth in Saccharomyces cerevisiae — Supporting Information 

# Relationships of RNA Polymerase II Genetic Interactors to Transcription Start Site Usage Defects and Growth in *Saccharomyces cerevisiae*

## Supporting Information for Jin and Kaplan, 2015

**Files in this Data Supplement:**

- Supporting Information - Figures S1-S6 and Table S1 (PDF, 846 KB)
- Figure S1 - Pol II/GTF double mutant effects on *ADH1* transcription start site selection. (PDF, 433 KB)
- Figure S2 - Phenotypes of *tfg2* alleles in combination with Pol II alleles. (PDF, 517 KB)
- Figure S3 - Phenotypes of *sua7* alleles in combination with Pol II alleles. (PDF, 574 KB)
- Figure S4 - Transcription start sites of genetic interactor deletions at *ADH1* detected by primer extension. (PDF, 365 KB)
- Figure S5 - Serial dilutions of Pol II genetic interactor deletions combined with *rpo21 (rpb1)* alleles to examine genetic interactions on general growth and on transcription-related gene-specific phenotypes (Spt-, MPAS, GalR phenotypes). (PDF, 584 KB)
- Figure S6 - Serial dilutions of WT and N1082S with an exogenous mutation (T1161R) to compare their phenotypes on growth media used in this study. (PDF, 343 KB)
- Table S1 - Yeast strains and plasmids used in this study. (PDF, 146 KB)
