# Supplementary material for: Relationships of RNA Polymerase II Genetic Interactors to Transcription Start Site Usage Defects and Growth in Saccharomyces cerevisiae
Source: G3 (Bethesda). 2014 Nov 6;5(1):21–33. doi: 10.1534/g3.114.015180 (PMC4291466; doi:10.1534/g3.114.015180)
Supplement: Supporting Information [file supp_g3.114.015180_015180SI.pdf]

G3 · Genes | Genomes | Genetics

Relationships of RNA Polymerase II genetic interactors to transcription start site usage defects and growth in *Saccharomyces cerevisiae*

Huiyan Jin* and Craig D. Kaplan*[1]

*Department of Biochemistry and Biophysics, Texas A&M University, College Station, TX 77843, USA

[1]Corresponding author: Craig D. Kaplan, 322A Biochemistry, Department of Biochemistry and Biophysics, Texas A&M University, College Station, TX 77843, USA. E-mail: cdkaplan@tamu.edu

DOI: 10.1534/g3.114.015180

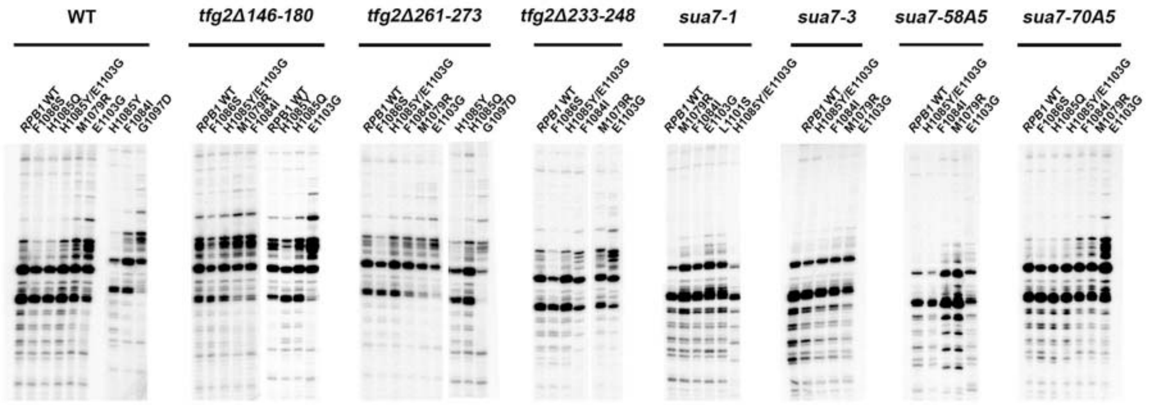

FIGURE S1 Pol II/GTF double mutant effects on *ADH1* transcription start site selection. TSSs at *ADH1* detected by primer extension for various transcription mutant strains. One representative experiment of at least three independent replicates is shown. GTF allele description is shown on top with a bar that indicates lanes showing Pol II alleles combined with a particular GTF allele. Relevant mutation in Pol II is labeled above each lane. Quantification of these experiments is shown in Figure 3.

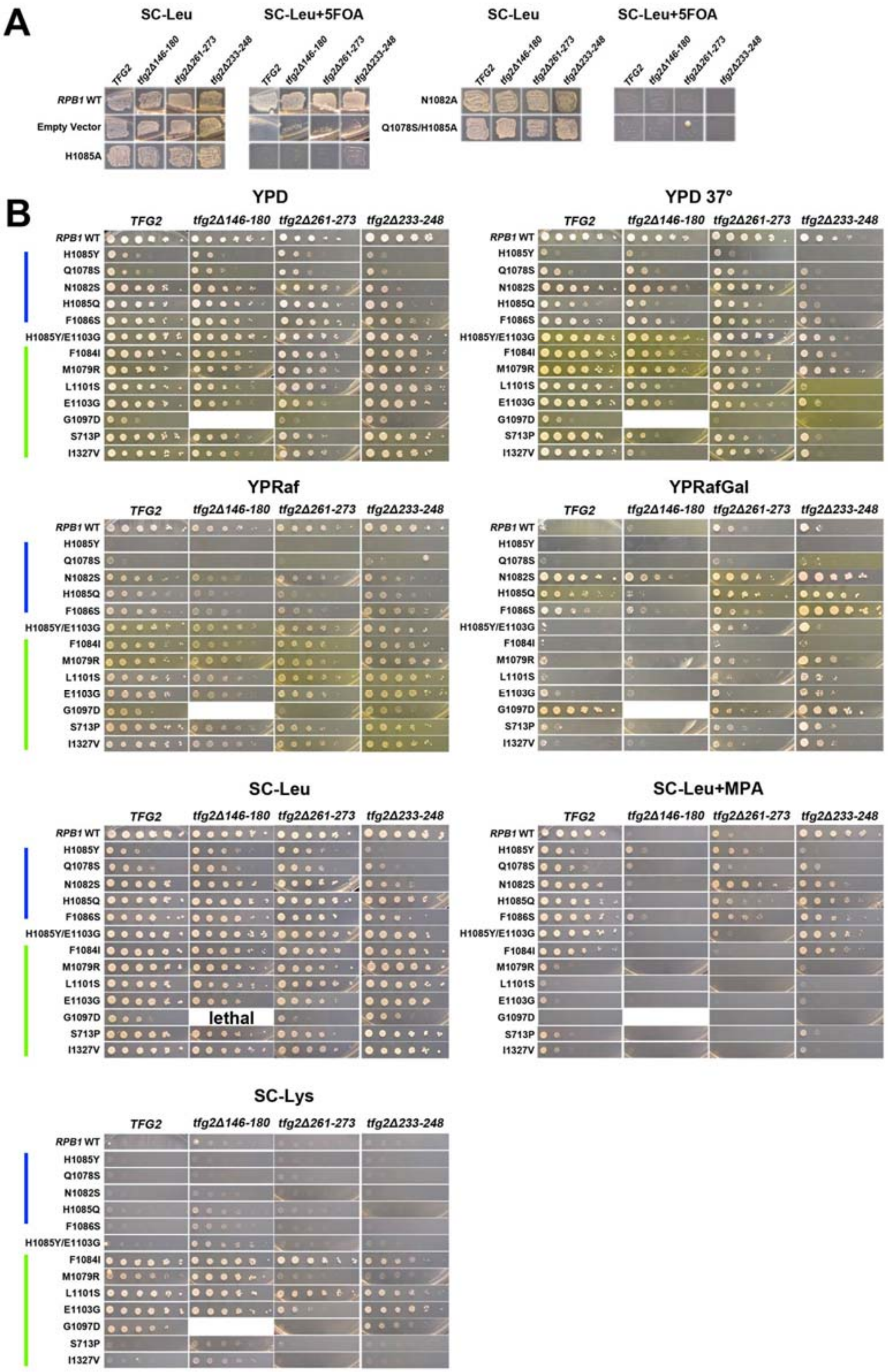

FIGURE S2 Phenotypes of *tfg2* alleles in combination with Pol II alleles. A. Inability of *tfg2* alleles to rescue lethal LOF Pol II alleles detected by growth of double mutants on 5FOA (see Methods and Materials for details). B. Serial dilutions of viable *tfg2*/*rpo21* (*rpb1*) double mutant alleles on various media for phenotyping of genetic interactions (general growth, temperature sensitivity, MPA^S, Gal^R and Spt^- phenotypes). LOF Pol II alleles are marked by blue bar, GOF by green. Heatmap presentation of phenotype quantifications of this assay is shown in Figure 2C.

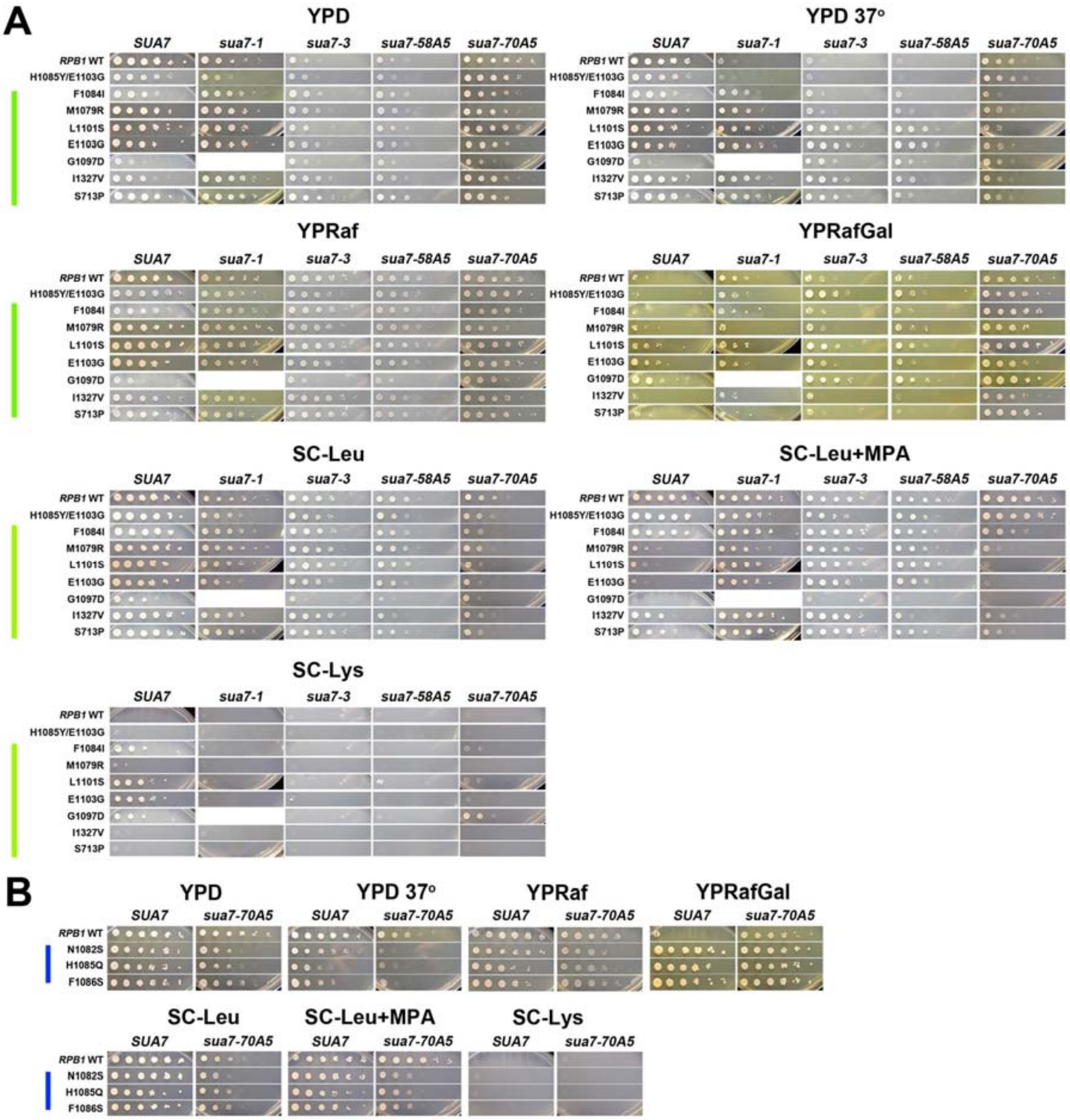

FIGURE S3 Phenotypes of *sua7* alleles in combination with Pol II alleles. A. Serial dilutions of viable *sua7*/*rpo21* (*rpb1*) double mutant alleles on various media for phenotyping of genetic interactions (general growth, temperature sensitivity, MPA^S, Gal^R and Spt$^-$ phenotypes). LOF Pol II alleles are marked by blue bar, GOF by green. Heatmap presentation of phenotype quantifications of this assay is shown in Figure 2D. B. Spot growth assay for combinations of LOF Pol II alleles with *sua7-70A5*.

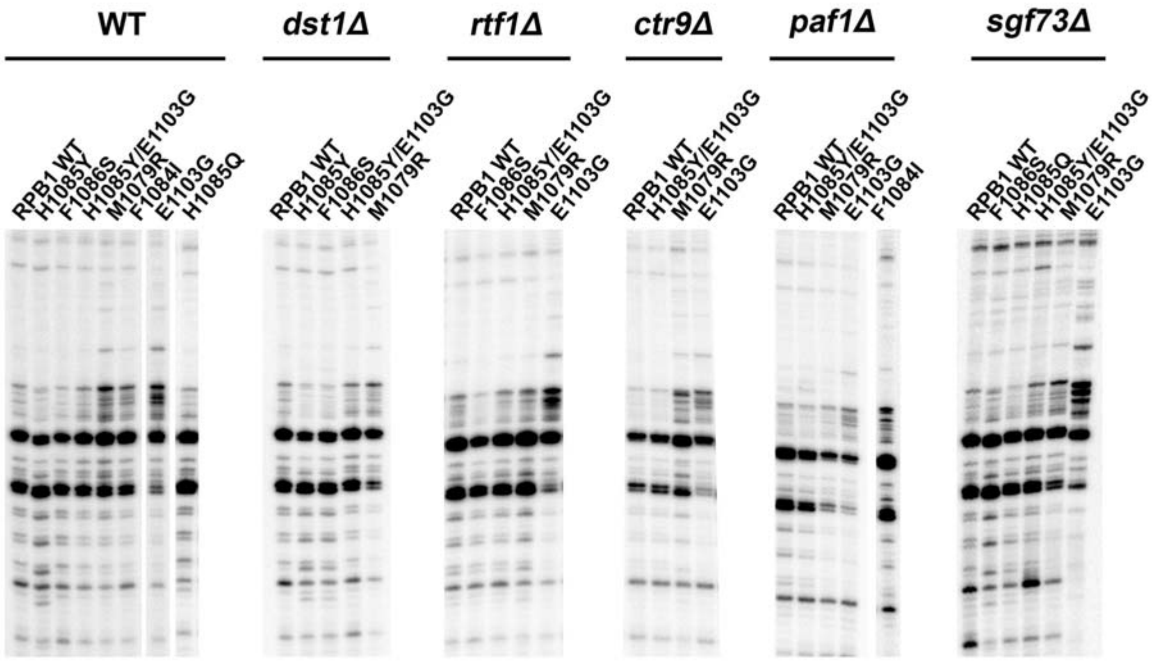

FIGURE S4 Transcription start sites of genetic interactor deletions at *ADH1* detected by primer extension. One representative experiment of at least three independent replicates is shown. Genetic interactor deletions are labeled above each group of relevant lanes for the double mutant combinations of a particular gene deletion mutant with Pol II alleles. Labels for relevant *rpo21/rpb1* genotypes are above each lane. Quantifications of these experiments are shown in Figure 5.

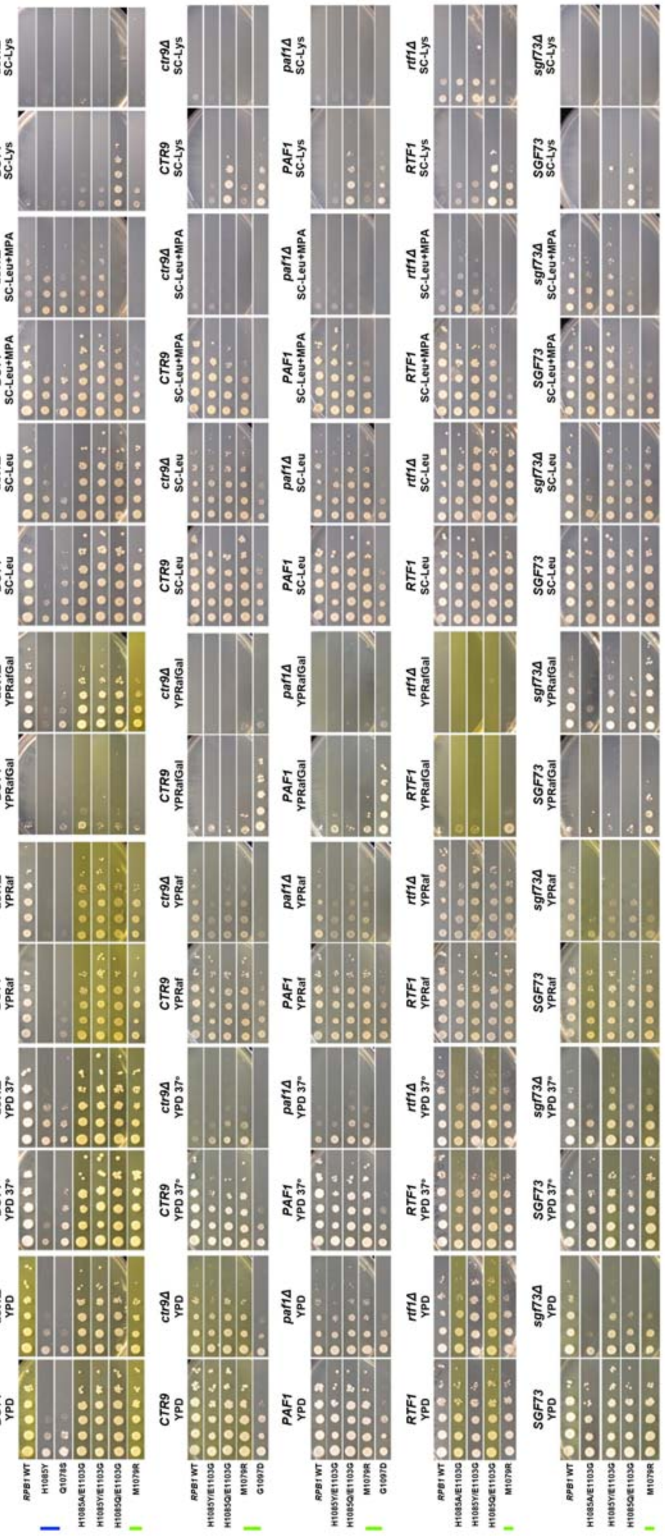

FIGURE S5 Serial dilutions of Pol II genetic interactor deletions combined with *rpo21 (rpb1)* alleles to examine genetic interactions on general growth and on transcription-related gene-specific phenotypes (Spt$^-$, MPA^S, Gal^R phenotypes). LOF Pol II alleles are marked by blue bar, GOF by green. Heatmap presentation of phenotype (general growth, temperature sensitivity, MPA^S, Gal^R and Spt$^-$ phenotypes) quantification of this assay is shown in Figure 4.

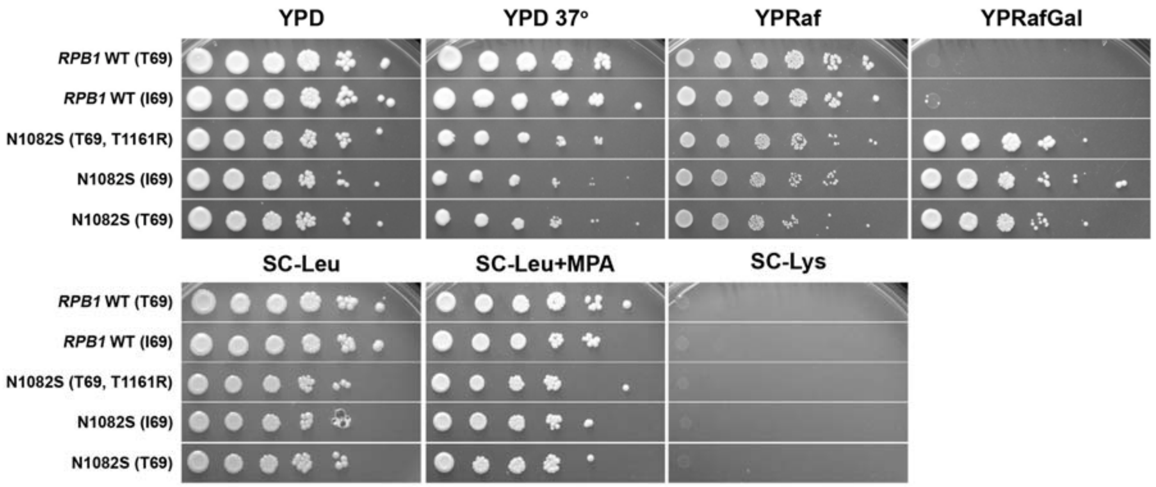

FIGURE S6 Serial dilutions of WT and N1082S with an exogenous mutation (T1161R) to compare their phenotypes on growth media used in this study. Different plasmids containing N1082S with and without extra mutations were tested on phenotyping media. Yeast strains containing N1082S along with T69I (a mutation found in the original cloned *RPO21* gene WT plasmid from the Young lab (described in Kaplan *et al.* 2012)) and/or T1161R (see note under Table S1) and corresponding *RPO21/RPB1* WT plasmids are shown. For more discussion of *rpo21/rpb1* T69I, see (Kaplan *et al.* 2012)

Table S1 Yeast strains and plasmids used in this study

Strain number	Relevant mutation	Genotype	Previous publication
CKY1529	*tfg2Δ146-180*	*MATa ura3-52 his3Δ200 leu2Δ1 or Δ0 trp1Δ63 met15Δ0 lys2-128∂ gal10Δ56 rpb1Δ::CLONATMX RPB3::TAP::KlacTRP1 tfg2Δ146-180 [pRP112 RPB1 CEN URA3]*	
CKY1530	*tfg2Δ233-248*	*MATa ura3-52 his3Δ200 leu2Δ1 or Δ0 trp1Δ63 met15Δ0 lys2-128∂ gal10Δ56 rpb1Δ::CLONATMX RPB3::TAP::KlacTRP1 tfg2Δ233-248 [pRP112 RPB1 CEN URA3]*	
CKY1531	*tfg2Δ261-273*	*MATa ura3-52 his3Δ200 leu2Δ1 or Δ0 trp1Δ63 met15Δ0 lys2-128∂ gal10Δ56 rpb1Δ::CLONATMX RPB3::TAP::KlacTRP1 tfg2Δ261-273 [pRP112 RPB1 CEN URA3]*	Braberg et al., 2013
CKY1543	*sua7-1*	*MATa ura3-52 his3Δ200 leu2Δ1 or Δ0 trp1Δ63 met15Δ0 lys2-128∂ gal10Δ56 rpb1Δ::CLONATMX RPB3::TAP::KlacTRP1 sua7-1 [pRP112 RPB1 CEN URA3]*	
CKY1544	*sua7-3*	*MATa ura3-52 his3Δ200 leu2Δ1 or Δ0 trp1Δ63 met15Δ0 lys2-128∂ gal10Δ56 rpb1Δ::CLONATMX RPB3::TAP::KlacTRP1 sua7-3 [pRP112 RPB1 CEN URA3]*	Braberg et al., 2013
CKY1545	*sua7-58A5*	*MATa ura3-52 his3Δ200 leu2Δ1 or Δ0 trp1Δ63 met15Δ0 lys2-128∂ gal10Δ56 rpb1Δ::CLONATMX RPB3::TAP::KlacTRP1 sua7-58A5 [pRP112 RPB1 CEN URA3]*	
CKY1546	*sua7-70A5*	*MATa ura3-52 his3Δ200 leu2Δ1 or Δ0 trp1Δ63 met15Δ0 lys2-128∂ gal10Δ56 rpb1Δ::CLONATMX RPB3::TAP::KlacTRP1 sua7-70A5 [pRP112 RPB1 CEN URA3]*	
CKY717	*dst1Δ*	*MATa ura3-52 his3Δ200 leu2Δ1 or Δ0 trp1Δ63 met15Δ0 lys2-128∂ gal10Δ56 rpb1Δ::CLONATMX RPB3::TAP::KlacTRP1 dst1Δ::KANMX [pRP112 RPB1 CEN URA3]*	Braberg et al., 2013
CKY740	*rtf1Δ*	*MATa ura3-52 his3Δ200 leu2Δ1 or Δ0 trp1Δ63 met15Δ0 lys2-128∂ gal10Δ56 rpb1Δ::CLONATMX RPB3::TAP::KlacTRP1 rtf1Δ::KANMX [pRP112 RPB1 CEN URA3]*	Braberg et al., 2013
CKY730	*ctr9Δ*	*MATa ura3-52 his3Δ200 leu2Δ1 or Δ0 trp1Δ63 met15Δ0 lys2-128∂ gal10Δ56 rpb1Δ::CLONATMX RPB3::TAP::KlacTRP1 ctr9Δ::KANMX [pRP112 RPB1 CEN URA3]*	Braberg et al., 2013
CKY729	*paf1Δ*	*MATa ura3-52 his3Δ200 leu2Δ1 or Δ0 trp1Δ63 met15Δ0 lys2-128∂ gal10Δ56 rpb1Δ::CLONATMX RPB3::TAP::KlacTRP1 paf1Δ::KANMX [pRP112 RPB1 CEN URA3]*	Braberg et al., 2013
CKY737	*sgf73Δ*	*MATa ura3-52 his3Δ200 leu2Δ1 or Δ0 trp1Δ63 met15Δ0 lys2-128∂ gal10Δ56 rpb1Δ::CLONATMX RPB3::TAP::KlacTRP1 sgf73Δ::KANMX [pRP112 RPB1 CEN URA3]*	Braberg et al., 2013

CKY283	WT	*MATa ura3-52 his3Δ200 leu2Δ1 or Δ0 trp1Δ63 met15Δ0 lys2-128∂ gal10Δ56 rpb1Δ::CLONATMX RPB3::TAP::KlacTRP1 [pRP112 RPB1 CEN URA3]*	Kaplan et al., 2012
**Plasmid number**	**Description**	**Genotype**	**Previous publication**
pCK1486	pRS306 *sua7-3*	*ampr, ColE1 ori URA3 sua7-3*	
pCK1487	pRS306 *sua7-1*	*ampr, ColE1 ori URA3 sua7-1*	
pCK1488	pRS306 *sua7-58A5*	*ampr, ColE1 ori URA3 sua7-58A5*	
pCK1489	pRS306 *sua7-70A5*	*ampr, ColE1 ori URA3 sua7-70A5*	
pCK1094	pRS306 *tfg2Δ146-180*	*ampr, ColE1 ori URA3 tfg2Δ146-180*	
pCK1096	pRS306 *tfg2Δ233-248*	*ampr, ColE1 ori URA3 tfg2Δ233-248*	
pCK1097	pRS306 *tfg2Δ261-273*	*ampr, ColE1 ori URA3 tfg2Δ261-273*	
pCK859	*RPB1* WT	*LEU2 CEN ARS ampr ColE1 ori RPB1*	Kaplan et al., 2012
pRS315	pRS315 empty vector	*LEU2 CEN ARS ampr ColE1 ori*	Sikorski and Hieter, 1989
pCK871	F1086S	*LEU2 CEN ARS ampr ColE1 ori rpb1 F1086S*	Kaplan et al., 2012
pCK887	H1085Q	*LEU2 CEN ARS ampr ColE1 ori rpb1 H1085Q*	Kaplan et al., 2012
pCK890	H1085Y/E1103G	*LEU2 CEN ARS ampr ColE1 ori rpb1 H1085Y/E1103G*	Kaplan et al., 2012
pCK899	H1085A/E1103G	*LEU2 CEN ARS ampr ColE1 ori rpb1 H1085A/E1103G*	Kaplan et al., 2012
pCK901	H1085Q/E1103G	*LEU2 CEN ARS ampr ColE1 ori rpb1 H1085Q/E1103G*	Kaplan et al., 2012
pCK872	M1079R	*LEU2 CEN ARS ampr ColE1 ori rpb1 M1079R*	Kaplan et al., 2012
pCK960	E1103G	*LEU2 CEN ARS ampr ColE1 ori rpb1 E1103G*	Kaplan et al., 2012
pCK955	F1084I	*LEU2 CEN ARS ampr ColE1 ori rpb1 F1084I*	Kaplan et al., 2012
pCK867	G1097D	*LEU2 CEN ARS ampr ColE1 ori rpb1 G1097D*	Kaplan et al., 2012
pCK886*	N1082S*	*LEU2 CEN ARS ampr ColE1 ori rpb1 N1082S T1161R*	Kaplan et al., 2012
pCK864	L1101S	*LEU2 CEN ARS ampr ColE1 ori rpb1 L1101S*	Kaplan et al., 2012
pCK528	S713P	*LEU2 CEN ARS ampr ColE1 ori rpb1 S713P I69*	Kaplan et al., 2012
pCK610	I1327V	*LEU2 CEN ARS ampr ColE1 ori rpb1 I1327V I69*	Kaplan et al., 2012
pCK347	RPB1 WT	*LEU2 CEN ARS ampr ColE1 ori RPB1 WT I69*	Kaplan et al., 2012
pCK638	N1082S	*LEU2 CEN ARS ampr ColE1 ori rpb1 N1082S T1161R I69*	
pCK1340	N1082S	*LEU2 CEN ARS ampr ColE1 ori rpb1 N1082S*	

*It was kindly brought to our attention by Tim Formosa (U. of Utah) that pCK886 *rpb1* N1082S *LEU2* also contains an additional mutation in *RPB1*, encoding T1161R. This mutation is outside of the trigger loop-encoding region and any region of this construct that was amplified using PCR (any DNA amplified by PCR for any construct is sequenced by default over the amplified region in the Kaplan lab). The spontaneous T1161R coding variant was not present in any parent or cousin plasmid to pCK886. We generated a corrected T1161 version of pCK886 N1082S and phenotyped it for all growth phenotypes examined in this manuscript (Figure S6). We observed no detectable differences in growth of N1082S/T1161R relative to N1082S/T1161 on any media tested and therefore conclude that T1161 is most likely phenotypically inert for these assays. Given the high level of sensitivity of our growth assays for detecting TSS-defective Pol II alleles, we conclude that T1161R does not likely modulate N1082S TSS defects.
