# Supplementary material for: Relationships of RNA Polymerase II Genetic Interactors to Transcription Start Site Usage Defects and Growth in Saccharomyces cerevisiae
Source: G3 (Bethesda). 2014 Nov 6;5(1):21–33. doi: 10.1534/g3.114.015180 (PMC4291466; doi:10.1534/g3.114.015180)
Supplement: Supporting Information [file supp_g3.114.015180_FigureS1.pdf]

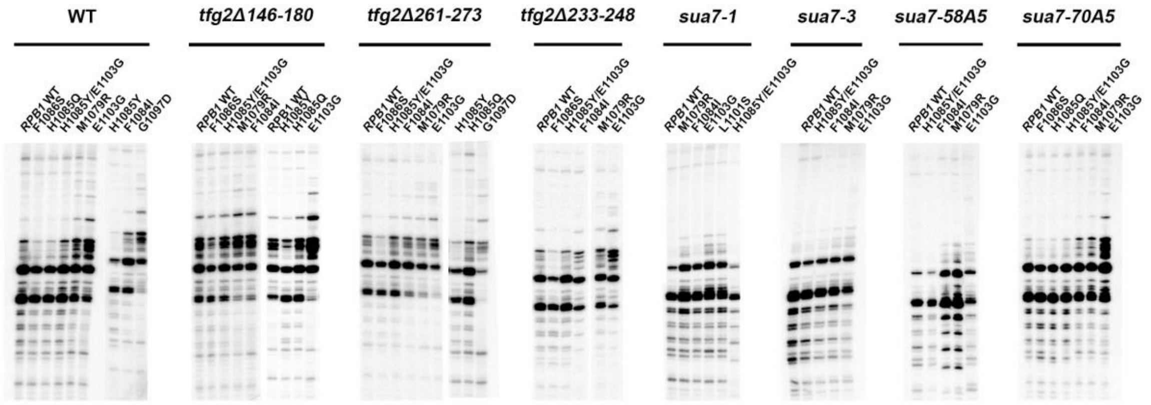

FIGURE S1 Pol II/GTF double mutant effects on *ADH1* transcription start site selection. TSSs at *ADH1* detected by primer extension for various transcription mutant strains. One representative experiment of at least three independent replicates is shown. GTF allele description is shown on top with a bar that indicates lanes showing Pol II alleles combined with a particular GTF allele. Relevant mutation in Pol II is labeled above each lane. Quantification of these experiments is shown in Figure 3.
