# Supplementary material for: Relationships of RNA Polymerase II Genetic Interactors to Transcription Start Site Usage Defects and Growth in Saccharomyces cerevisiae
Source: G3 (Bethesda). 2014 Nov 6;5(1):21–33. doi: 10.1534/g3.114.015180 (PMC4291466; doi:10.1534/g3.114.015180)
Supplement: Supporting Information [file supp_g3.114.015180_FigureS2.pdf]

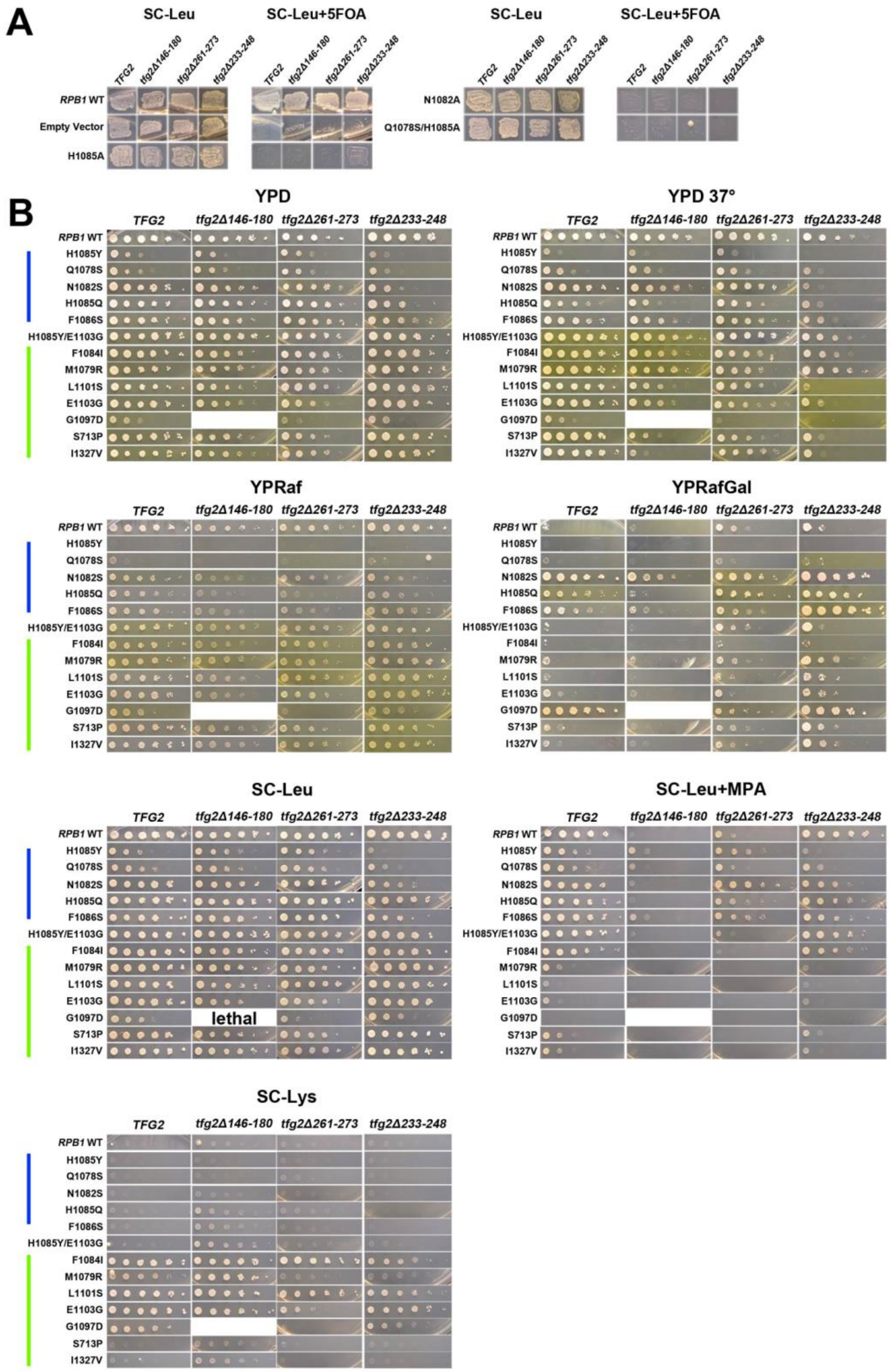

FIGURE S2 Phenotypes of *tfg2* alleles in combination with Pol II alleles. A. Inability of *tfg2* alleles to rescue lethal LOF Pol II alleles detected by growth of double mutants on 5FOA (see Methods and Materials for details). B. Serial dilutions of viable *tfg2*/*rpo21* (*rpb1*) double mutant alleles on various media for phenotyping of genetic interactions (general growth, temperature sensitivity, MPA^S, Gal^R and Spt$^-$ phenotypes). LOF Pol II alleles are marked by blue bar, GOF by green. Heatmap presentation of phenotype quantifications of this assay is shown in Figure 2C.
