# Supplementary material for: Relationships of RNA Polymerase II Genetic Interactors to Transcription Start Site Usage Defects and Growth in Saccharomyces cerevisiae
Source: G3 (Bethesda). 2014 Nov 6;5(1):21–33. doi: 10.1534/g3.114.015180 (PMC4291466; doi:10.1534/g3.114.015180)
Supplement: Supporting Information [file supp_g3.114.015180_FigureS3.pdf]

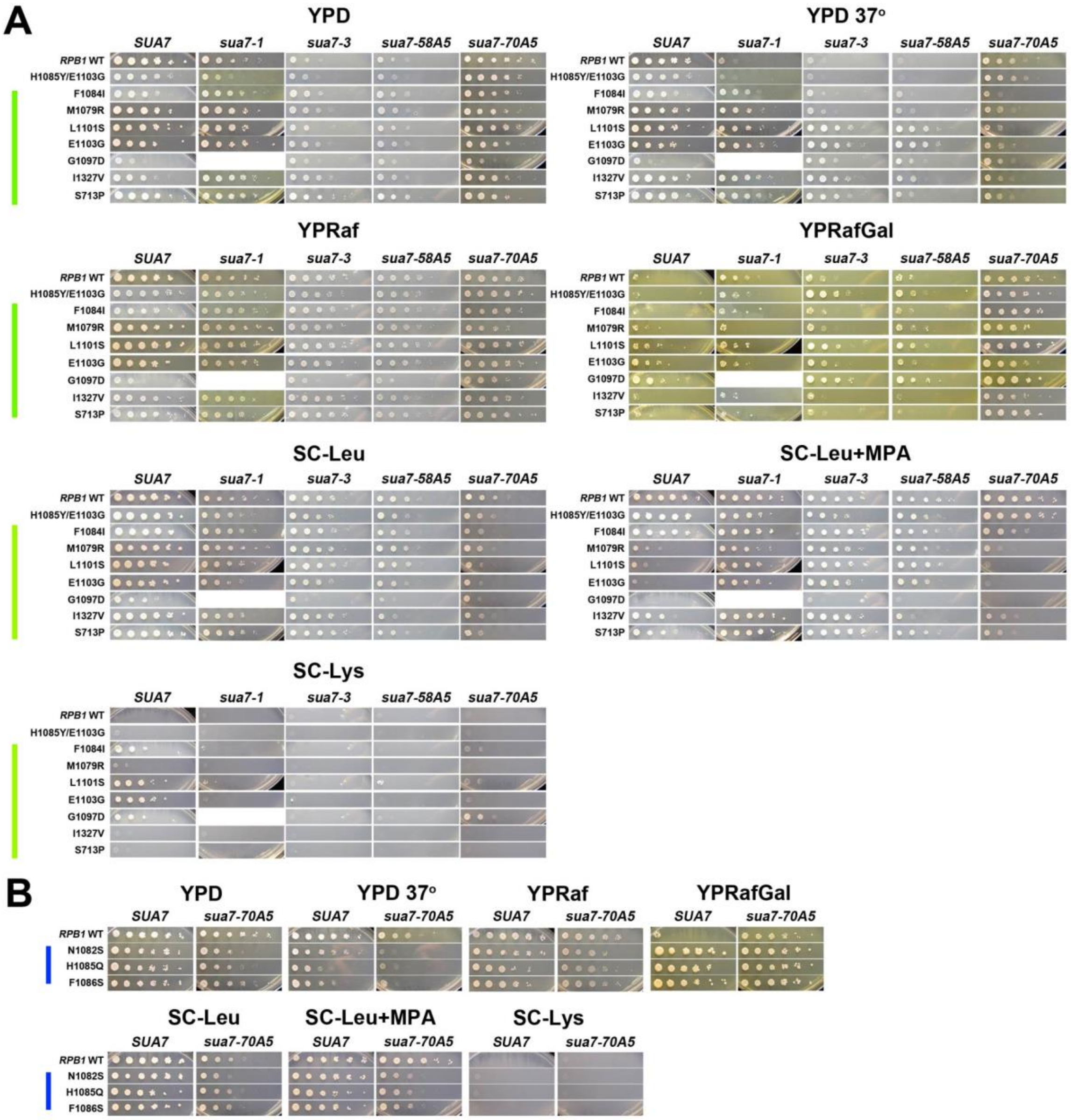

FIGURE S3 Phenotypes of *sua7* alleles in combination with Pol II alleles. A. Serial dilutions of viable *sua7*/*rpo21* (*rpb1*) double mutant alleles on various media for phenotyping of genetic interactions (general growth, temperature sensitivity, MPA^S, Gal^R and Spt$^-$ phenotypes). LOF Pol II alleles are marked by blue bar, GOF by green. Heatmap presentation of phenotype quantifications of this assay is shown in Figure 2D. B. Spot growth assay for combinations of LOF Pol II alleles with *sua7-70A5*.
