# Supplementary material for: Relationships of RNA Polymerase II Genetic Interactors to Transcription Start Site Usage Defects and Growth in Saccharomyces cerevisiae
Source: G3 (Bethesda). 2014 Nov 6;5(1):21–33. doi: 10.1534/g3.114.015180 (PMC4291466; doi:10.1534/g3.114.015180)
Supplement: Supporting Information [file supp_g3.114.015180_FigureS4.pdf]

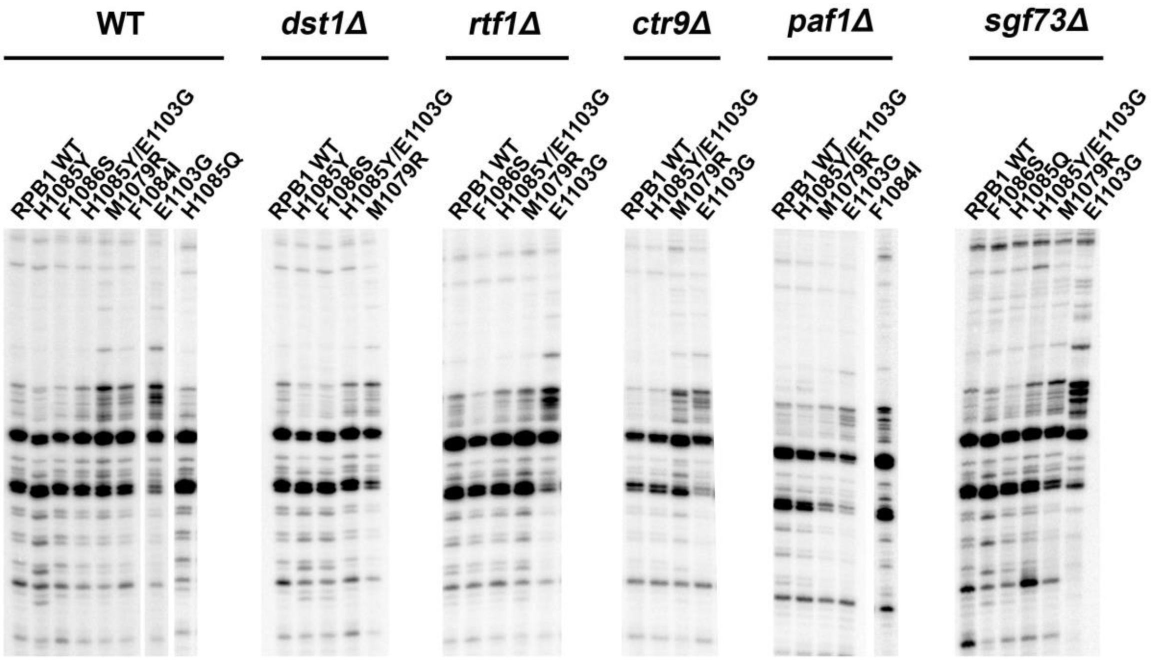


**FIGURE S4** Transcription start sites of genetic interactor deletions at *ADH1* detected by primer extension. One representative experiment of at least three independent replicates is shown. Genetic interactor deletions are labeled above each group of relevant lanes for the double mutant combinations of a particular gene deletion mutant with Pol II alleles. Labels for relevant *rpo21/rpb1* genotypes are above each lane. Quantifications of these experiments are shown in Figure 5.
