# Supplementary material for: Relationships of RNA Polymerase II Genetic Interactors to Transcription Start Site Usage Defects and Growth in Saccharomyces cerevisiae
Source: G3 (Bethesda). 2014 Nov 6;5(1):21–33. doi: 10.1534/g3.114.015180 (PMC4291466; doi:10.1534/g3.114.015180)
Supplement: Supporting Information [file supp_g3.114.015180_FigureS5.pdf]

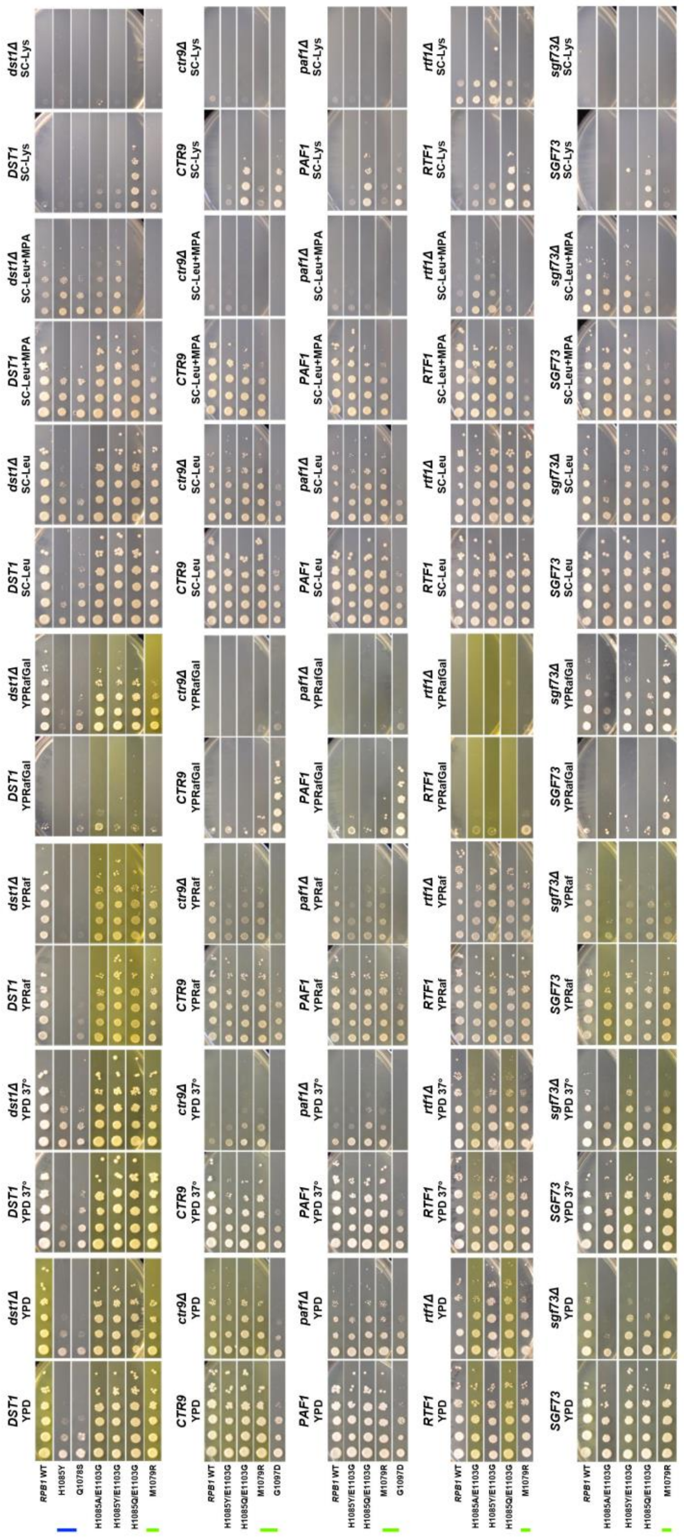

FIGURE S5 Serial dilutions of Pol II genetic interactor deletions combined with *rpo21 (rpb1)* alleles to examine genetic interactions on general growth and on transcription-related gene-specific phenotypes (Spt$^-$, MPA^S, Gal^R phenotypes). LOF Pol II alleles are marked by blue bar, GOF by green. Heatmap presentation of phenotype (general growth, temperature sensitivity, MPA^S, Gal^R and Spt$^-$ phenotypes) quantification of this assay is shown in Figure 4.
