# Supplementary material for: Relationships of RNA Polymerase II Genetic Interactors to Transcription Start Site Usage Defects and Growth in Saccharomyces cerevisiae
Source: G3 (Bethesda). 2014 Nov 6;5(1):21–33. doi: 10.1534/g3.114.015180 (PMC4291466; doi:10.1534/g3.114.015180)
Supplement: Supporting Information [file supp_g3.114.015180_FigureS6.pdf]

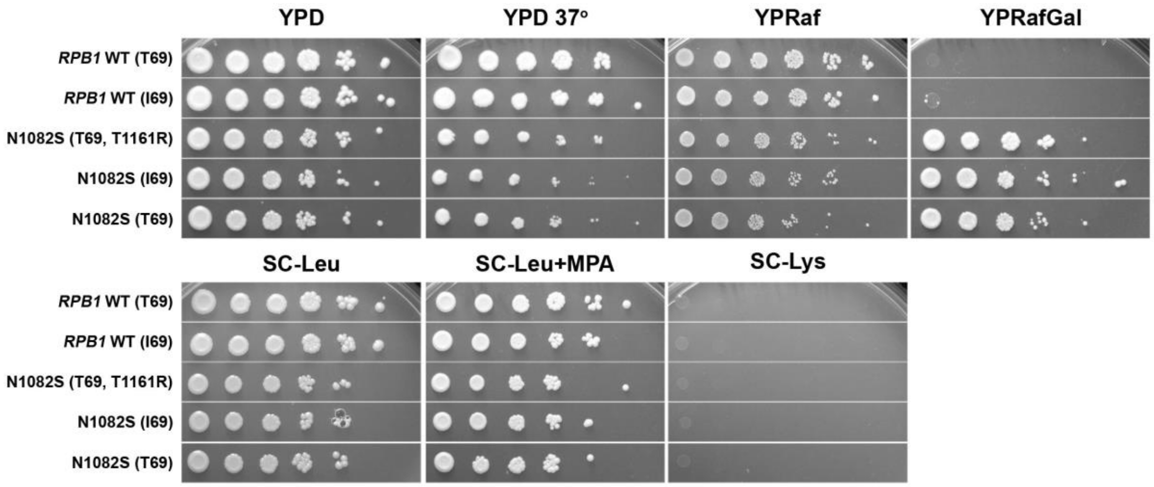

FIGURE S6 Serial dilutions of WT and N1082S with an exogenous mutation (T1161R) to compare their phenotypes on growth media used in this study. Different plasmids containing N1082S with and without extra mutations were tested on phenotyping media. Yeast strains containing N1082S along with T69I (a mutation found in the original cloned *RPO21* gene WT plasmid from the Young lab (described in KAPLAN *et al.* 2012)) and/or T1161R (see note under Table S1) and corresponding *RPO21/RPB1* WT plasmids are shown. For more discussion of *rpo21/rpb1* T69I, see (KAPLAN *et al.* 2012)
